# Pharmaco-toxicological effects of the novel tryptamine hallucinogen 5-MeO-MiPT on motor, sensorimotor, physiological, and cardiorespiratory parameters in mice—from a human poisoning case to the preclinical evidence

**DOI:** 10.1007/s00213-024-06526-8

**Published:** 2024-01-12

**Authors:** Marta Bassi, Sabrine Bilel, Micaela Tirri, Giorgia Corli, Fabiana Di Rosa, Adolfo Gregori, Alaaldin M. Alkilany, Ousama Rachid, Elisa Roda, Giorgio Zauli, Carlo Alessandro Locatelli, Matteo Marti

**Affiliations:** 1 Department of Translational Medicine, Section of Legal Medicine and LTTA Centre, University of Ferrara, Via Fossato Di Mortara 70, 44121 Ferrara, Italy; 2 Department of Scientific Investigation (RIS), Carabinieri, 00191 Rome, Italy; 3 Department of Pharmaceutical Sciences, College of Pharmacy, QU Health, Qatar University, Doha, Qatar; 4 Laboratory of Clinical & Experimental Toxicology, Pavia Poison Centre, National Toxicology Information Centre, Toxicology Unit, Istituti Clinici Scientifici Maugeri, IRCCS, Pavia, Italy; 5 Research Department, King Khaled Eye Specialistic Hospital, Riyadh, Saudi Arabia; 6 Department of Anti-Drug Policies, Collaborative Center for the Italian National Early Warning System, Presidency of the Council of Ministers, Ferrara, Italy

**Keywords:** 5-MeO-MiPT, Human intoxication, Serotoninergic hallucinogens, Behaviour, Cardiorespiratory changes, Prepulse inhibition, ADMET prediction

## Abstract

**Rationale:**

The 5-methoxy-N-methyl-N-isopropyltryptamine (5-MeO-MiPT, known online as “Moxy”) is a new psychedelic tryptamine first identified on Italian national territory in 2014. Its hallucinogen effects are broadly well-known; however, only few information is available regarding its pharmaco-toxicological effects.

**Objectives:**

Following the seizure of this new psychoactive substances by the Arm of Carabinieri and the occurrence of a human intoxication case, in the current study we had the aim to characterize the in vivo acute effects of systemic administration of 5-MeO-MiPT (0.01–30 mg/kg i.p.) on sensorimotor (visual, acoustic, and overall tactile) responses, thermoregulation, and stimulated motor activity (drag and accelerod test) in CD-1 male mice. We also evaluated variation on sensory gating (PPI, prepulse inhibition; 0.01–10 mg/kg i.p.) and on cardiorespiratory parameters (MouseOx and BP-2000; 30 mg/kg i.p.). Lastly, we investigated the in silico ADMET (absorption, distribution, metabolism, excretion, toxicity) profile of 5-MeO-MiPT compared to 5-methoxy-N,N-diisopropyltryptamine (5-MeO-DIPT) and N,N-dimethyltryptamine (DMT).

**Results:**

This study demonstrates that 5-MeO-MiPT dose-dependently inhibits sensorimotor and PPI responses and, at high doses, induces impairment of the stimulated motor activity and cardiorespiratory changes in mice. In silico prediction shows that the 5-MeO-MiPT toxicokinetic profile shares similarities with 5-MeO-DIPT and DMT and highlights a cytochrome risk associated with this compound.

**Conclusions:**

Consumption of 5-MeO-MiPT can affect the ability to perform activities and pose a risk to human health status, as the correspondence between the effects induced in mice and the symptoms occurred in the intoxication case suggests. However, our findings suggest that 5-MeO-MiPT should not be excluded from research in the psychiatric therapy field.

## Introduction

**Fig. 1:**
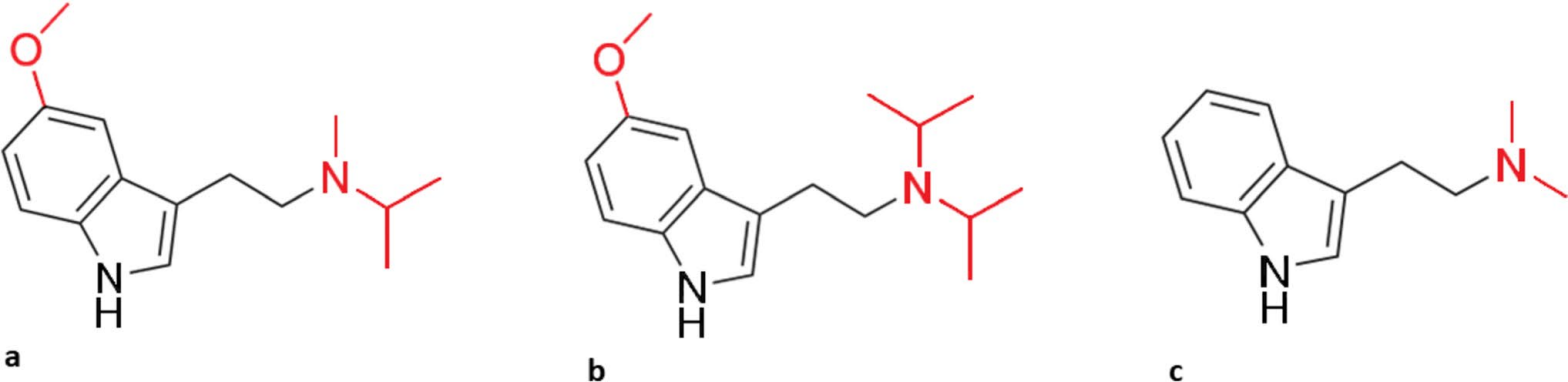
Chemical structure of **a** 5-methoxy-N-methyl-N-isopropyltryptamine (5-MeO-MiPT); **b** 5-methoxy-N,N-diisopropyltryptamine (5-MeO-DIPT); and **c** N,N-dimethyltryptamine (DMT)

The synthesis and the spread of new psychoactive substances (NPS) are a growing phenomenon that constantly pose challenges for law enforcements, clinicians, and global public health. Indeed, in 2020 more than 350 laboratories were dismantled with almost 7 tons of NPS seized and 880 NPS monitored (EMCDDA 2021, 2022). Despite hallucinogens represent a class of drugs with a prevalence of use in Europe generally low compared to others, the European Monitoring Centre for Drugs and Drug Addiction (EMCDDA) reported that 163 new psychedelics were monitored in 2021 (57 tryptamines and 106 phenethylamines; EMCDDA 2022). Moreover, between 2022 and 2023, 82 countries reported a total of 167 classic hallucinogens, representing 14% of the NPS registered (UNODC 2023). Nowadays psychedelics are divided into three groups based on their chemical structure (Baumeister et al. 2015): lysergamides (including LSD and its analogues), tryptamines (including psilocin and its analogues), and phenethylamines (including mescaline and its analogues). These substances alter perception, thought, and state of consciousness acting on serotonin (5HT) receptor 2A (5HT_2A_; Halberstadt 2015), with a possible interaction with other serotonin receptor subtypes such as 1A (5HT_1A_) and 2C (Halberstadt and Geyer 2011; Zamberlan et al. 2018). Regarding tryptamines, the core structure contains an indole ring linked by an ethyl side chain to an amino group, which can be mono- or di-substituted by alkyl groups, and the indole ring can support substitution on position 4 or 5 (Fantegrossi et al. 2008). These compounds present a non-selective action on 5HT receptors with quite different potency and affinity profiles. It has been shown that the 4-hydroxylation or the 5-methoxylation is associated with increased potency in comparison to the analogues with different substitutions (Araújo et al. 2015). Some of them, like N,N-dimethyltryptamine (DMT; Fig. 1c) and derivates, bind adrenergic-α1 receptors and are substrate for SERT (serotonin transporter; Halberstadt 2015). They bind various targets in vitro, including dopaminergic, adrenergic, and histaminergic receptors, as well as monoaminergic transporters (Luethi and Liechti 2020). Tryptamines are generally consumed with the aim to alter perception but can induce psychological disturbance, including psychosis. Adverse effects of these compounds are restlessness, disorientation, confusion, hallucinate state, amnesia, mydriasis, tachypnoea, hypertension, and tachycardia. Cases of acute renal failure and rhabdomyolysis associated with synthetic tryptamine intoxications have been reported, as well as deaths (Luethi and Liechti 2020). From 2009 to 2013, eight tryptamines were intercepted on Italian territory (Serpelloni et al. 2013) and during 2013 the Italian National Early Warning System (SNAP) reported the seizure of tryptamines, such as 5-MeO-DALT, 4-AcO-DMT, and DET (Allerta Dronetplus 2016).

The 5-methoxy-N-methyl-N-isopropyltryptamine (5-MeO-MiPT; Fig. 1a), also known as “Moxy” in the illicit drug market, is an analogue of 5-methoxy-N,N-diisopropyltryptamine (5-MeO-DIPT, “Foxy”; Fig. 1b; Altuncı et al. 2021). Its synthesis was first reported by Repke et al. (1985) and its psychoactive effects were first documented by Shulgin and Shulgin (1997). This analogue has been involved in cases of intoxications in Japan (Itowaka et al. 2007; Shimizu et al. 2007) and in a case of homicide as a result of a sever psychotic state induced by the simultaneous intake of 5-MeO-MiPT and 5-MeO-DIPT (Matsumoto and Okada 2006). In 2017, a suicide associated to 3-MeO-PCP has been reported, in which post-mortem analysis revealed the presence of 5-MeO-MiPT (concentration of 0.13 µg/g in femoral blood; Johansson et al. 2017). The compound was first reported to the National Early Warning System (SNAP) in November 2013 and has been identified for the first time on Italian territory in 2014 (Dipartimento Politiche Antidroga 2015). In fact, on 18 May 2018 5-MeO-MiPT has been included in Table I of narcotic and psychotropic substances (DPR 309/90). The compound is available online in the form of powder or liquid and it can be orally consumed (4–6 mg) or inhaled (12–20 mg; Erowid 2015a, b, c; Tittarelli et al. 2015). According to users’ report, the psychoactive effect following the oral ingestion appears after 15–20 min with a peak approximately between 45 and 60 min and seems to decrease in 10 h. On the other hand, if inhaled the onset is immediate and the offset is about 2–5 h later. The effects reported by users vary from sleepy to speedy and from sense enhancing to mind-numbing (Erowid 2015a, b, c). In particular, it induces a full range of high-level hallucinatory states, visual distortions, colour and visual acuity enhancement, enhanced sense of touch, relaxation, and sleep disturbances (Tittarelli et al. 2015; Psychonautwiki 2023a, b). Adverse effects described are stomach discomfort, gas and vomiting, anxious stimulation, and muscle tension (Erowid 2015a, b, c). This is in line with symptoms associated with its analogue 5-MeO-DIPT and DMT, which induce stomach discomfort, nausea and vomiting, disorientation, tachycardia, hypertension, confusion, headache, and speech difficulties, basing on information reported by consumers (Erowid 2015a, b, c; Psychonautwiki 2022, 2023a, b) and case reports (Smolinske et al. 2005; Muller 2004; Heise and Brooks 2017; Bouso et al. 2022). In vitro studies by Rickli and colleagues showed that 5-MeO-MIPT is a full agonist of 5HT_2A_ receptor (activation efficacy up to 80%) with a value of EC50 of 0.023 ± 0.04 µM, but it presents a higher affinity with 5HT_1A_ receptor than with 5HT_2A_ (values of Ki respectively of 0.058 ± 0.01 µM and 0.163 ± 0.03 µM; Rickli et al. 2016). Concerning interaction with other targets, it has been found that 5-MeO-MiPT inhibited serotonin and norepinephrine uptake in rats (Nagai et al. 2007), but in contrast with these results another study showed that the substance does not interact with rat monoamine transporters (Blough et al. 2014).

In 2021, the Arm of Carabinieri seized 5-MeO-MIPT in a form of powder and, in 2022, the Pavia Poison Control Centre (PCC)—National Toxicology Information Centre alerted our laboratory about a non-fatal human intoxication involving 5-MeO-MiPT. Both these events underlined the urgent need to expand the knowledge on the pharmaco-toxicology of this tryptamine analogue. Indeed, as a collaborative centre for the Italian National Early Warning System, committed to the monitoring and prevention of NPS abuse, we intended to characterize the toxicological effects of this above reported NPS through our preclinical investigations.

With this purpose, the current study aimed to evaluate the in vivo acute effects of 5-MeO-MIPT on neurological, sensorimotor (visual, tactile, and acoustic), motor activity, and thermoregulation, together with the possible alteration of the sensorimotor gating system through PPI (prepulse inhibition) in murine model. Also, we investigated the effects on cardiovascular and respiratory parameters in mice. Finally, we evaluated in silico ADMET (absorption, distribution, metabolism, excretion, toxicity) profile of 5-MeO-MiPT in comparison to 5-MeO-DIPT and DMT to provide a rapid screening of the toxic potential of these compounds.

## Materials and methods

### Animals

A total of 110 adult male ICR (CD-1®) mice, 3–4 months old, weighing 25–30 g (ENVIGO Harlan Italy, Italy; bred inside the Laboratory for Preclinical Research, LARP, of University of Ferrara, Italy), were group-housed (5 mice per cage; a floor area per animal of 80 cm^2^; a minimum enclosure height of 12 cm on a 12:12-h light–dark cycle (light on at 6:30 AM), the temperature of 20–22 °C, the humidity of 45–55%) and were provided ad libitum access to food (Diet 4RF25 GLP; Mucedola, Settimo Milanese, Milan, Italy) and water. Experiments were performed during the light phase of the light–dark cycle (lights on between 6:30 AM and 6:30 PM). The experimental protocols followed in the present study were in accordance with the UK Animals (Scientific Procedures) Act of 1986 and associated guidelines and the new European Communities Council Directive of September 2010 (2010/63/EU). The Italian Ministry of Health (licence n. 223/2021-PR and extension CBCC2.46.EXT.21) and the Animal Welfare Body of the University of Ferrara approved experimental protocols. According to the ARRIVE guidelines, all possible efforts were made to minimize the number of animals used, to minimize the animals’ pain and discomfort. For the present study, mice were divided into different groups, as follows. In safety pharmacology studies (visual, acoustic, and tactile sensorimotor responses; body core and surface temperature; accelerod and drag tests), eight mice (total of 48 mice) were employed for each treatment (vehicle or five doses: 0.01, 0.1, 1, 10, and 30 mg/kg). In the PPI test, eight mice (total of 40 mice) were used for each treatment (vehicle or four doses: 0.01, 0.1, 1, and 10 mg/kg). In cardiorespiratory studies, six mice (total of 12 mice) were utilized for each treatment (vehicle or 30 mg/kg). In blood pressure studies, five mice (total of 10 mice) were employed for each treatment (vehicle or 30 mg/kg).

As indicated by the latest European School Survey Project on Alcohol and Other Drugs report (ESPAD 2020), the lifetime prevalence of use of hallucinogens is higher in male (2.4%) than female (1.7%) population. Moreover, the majority of intoxications with designer tryptamines reported in literature concerns male subjects (Muller 2004; Smolinske et al. 2005; Matsumoto and Okada 2006; Shimizu et al. 2007; Johansson et al. 2017; Bilhimer et al. 2018). Furthermore, the intoxication case reported by the Pavia Poison Control Centre (PCC)—National Toxicology Information Centre involved a 23-year-old male. All these observations led us to investigate the effects of 5-MeO-MiPT at first in male subjects. However, we are aware that abuse of those compounds can as well involve female population, as the case reported by Itowaka and colleagues suggests (Itokawa et al. 2007). Moreover, it is known that gender and sex can importantly affect the responses to drugs (Fattore et al. 2020). In this regard, such studies have been conducted on murine model to evaluate any sex-related differences after hallucinogens administration. For example, female rats were reported to be more sensitive than males to the effects of 5-MeO-DMT (Shephard and Broadhurst 1983), while other studies stated that females are lesser affected than males by behavioural changes induced by LSD (Pálenícek et al. 2010) and psilocin (Tylš et al. 2016). Basing on this evidence, it can be assumed that male mice could be more sensitive to the effects of 5-MeO-MiPT on locomotor activity and sensory gating alterations than females. However, due to the higher sensitivity of females to the effect on hyponeophagia induced by 5-MeO-DMT showed by Shephard and Broadhurst, further research must be conducted to clarify this conflicting aspect.

### Drug preparation and dose selection

The 5-MeO-MiPT was provided by the Forensic Science Department of Arm of Carabinieri (Roma’s R.I.S., referent Colonel Adolfo Gregori). The drug was dissolved in Tween 80 (2%) and ethanol (5%), brought to the final volume with saline (0.9% NaCl), and administered by intraperitoneal (i.p.) injection at a volume of 4 µL/g. Tween 80 (2%), ethanol (5%), and saline were also used as the vehicle. Doses of 5-MeO-MiPT were chosen based on the behavioural and neurological effects reported on internet experiences with the compound (Erowid 2015a, b, c) and other tryptamine hallucinogens (5-MeO-DIPT, 5-MeO-DMT, DMT). The human doses were converted to animal doses using the body surface area (BSA) normalization method (Reagan-Shaw et al. 2008). In particular, the lower doses (0.01 and 0.1 mg/kg; human equivalent doses of 0.00081 mg/kg and 0.0081 mg/kg in a 60 kg subject) tested in this study are equivalent to a human subthreshold dose, the intermediate dosage (1 mg/kg; human equivalent dose of 0.081 mg/kg) tested amounts to a common human oral dose, while the higher dosages (10 and 30 mg/kg; human equivalent doses of 0.81 and 2.43 mg/kg) tested exceed the highest values consumed by users (Psychonautwiki 2023a, b). Regarding PPI test, basing on our previous preclinical studies on hallucinogens (Tirri et al. 2022) and preliminary data, we decided to use a different range of doses (0.01–10 mg/kg). Instead, in cardiorespiratory and blood pressure tests we injected the highest dose of 5-MeO-MiPT (30 mg/kg) to simulate the heaviest intoxication.

### Behavioural tests

The effect of 5-MeO-MiPT were investigated using a battery of behavioural tests widely employed in our laboratory for safety pharmacology studies for the preclinical characterization of NPS in rodents (Bilel et al. 2020, 2023; Corli et al. 2023). Mice were evaluated using different tests carried out in a consecutive manner, as follows. Observation of visual object (frontal and lateral) and placing responses, acoustic and tactile (pinna, vibrissae, and corneal reflexes) responses, were measured 10, 30, 60, 120, 180, 240, and 300 min after injection of the compound. The body core and surface temperatures were measured 15, 35, 65, 125, 185, 245, and 305 min after treatment. Accelerod test was measured at 15, 40, 70, 130, 190, 250, and 310 min, while drag test was measured at 45, 70, 105, 160, 220, 280, and 340 min. For the visual object, acoustic, and tactile sensorimotor tests, each mouse was housed in an experimental chamber (350 × 350 × 350 mm) made with black methacrylate walls and a transparent front door. A camera (B/W USB Camera day and night with varifocal lens; Ugo Basile, Italy) was placed at the top and/or side of the box. For the evaluation of effects of 5-MeO-MiPT on acoustic sensorimotor gating, the startle/PPI technique (Ugo Basile apparatus, Milan, Italy) was used. Behavioural tests were conducted in the Centralized Preclinical Research Laboratory of University of Ferrara at a thermostated temperature of 20–22 °C, humidity of approximately 45–55%, and controlled light (approximately 150 lx) with a background noise of approximately 40 ± 4 dB. All experiments were performed between 8:30 AM and 2:00 PM and conducted blindly by trained observers working in pairs.

#### Evaluation of visual response (object and placing response tests)

Visual response was measured by two behavioural tests, which evaluated the ability of the mouse to capture visual information when it is stationary (visual object response) or moving (visual placing response). The visual object response test consists of approaching an object (respectively a white horizontal bar and a small dentist’s mirror) from the front (frontal view) or the side (lateral view) to the mouse and evaluate the animal ability to see it, if when the mouse typically shifts or turns the head, or retreat (Ossato et al. 2015; Marti et al. 2019; Bilel et al. 2020, 2023). Both frontal and lateral visual response tests were repeated three times. The assigned score was 1 if there was a reflection in the mouse movement, or 0 if it was not present. The total value was calculated by adding the single scores of frontal and lateral visual response tests (overall score: 9).

Visual placing response test was performed using a tail suspension modified apparatus able to bring down the mouse towards the floor at a constant speed of 10 cm/s (Ossato et al. 2015). Mice were suspended 20 cm above the floor by an adhesive tape that was placed approximately 1 cm from the tip of the tail. The downward movement of the mouse was videotaped by a camera (B/W USB Camera day and night with varifocal lens; Ugo Basile, Italy) placed at the base of the tail suspension apparatus. Movies were analysed off-line by a trained operator who did not know the drug treatments performed. The analysis frame by frame allowed to evaluate the beginning of the reaction of the mouse while it was close to the floor. The first movement of the mouse when it perceives the floor is the extension of the front legs. When the mouse started the reaction, an electronic ruler evaluated the perpendicular distance in millimetres between the first-mice movement reaction and the floor.

#### Evaluation of overall tactile and acoustic responses

Tactile response of the mice was verified through vibrissae, corneal, and pinnae reflexes (Ossato et al. 2015). Vibrissae reflex was evaluated by touching vibrissae (right and left) with a gavage needle once for side, a score of 1 was given if there was a response (the mouse turns the head to the side of touch or vibrissae movement), and a score of 0 was given if there was no response (overall score: 2). Corneal reflex was assessed gently touching the cornea of the mouse with a gavage needle and evaluating the response, assigning a value of 1 if the mouse responded closing the eyelid and a value of 0 if there was no response. The test was repeated 3 times and was conducted bilaterally (overall score: 6). Pinna reflex was assessed by touching pavilions (left and right) with a gavage needle, first the interior and then the external pavilions. The test is repeated twice for side giving a value of 1 if there was a reflex and 0 if not present (overall score: 4). Data are expressed as the sum of the three parameters (overall score: 12).

Evaluation of acoustic response measures the reflexes of the mouse in response to an acoustic stimulus produced behind the animal (Ossato et al. 2015). Four acoustic stimuli of different intensities and frequencies were tested: a snap of the fingers, a sharp click (produced by a metal instrument), an acute (frequency of about 5.0–5.1 kHz, produced by an audiometer), and a sever (frequency of about 125–150 kHz, produced by an audiometer). Each sound was repeated three times, a score of 1 was given if there was a response, and a score of 0 was given if there was no response (total score of 3 for each sound). The acoustic total score was calculated by adding scores obtained in the four tests (overall score: 12).

#### Evaluation of core temperature and body surface temperature

The core temperature was evaluated by a probe (1 mm diameter) that was gently inserted, after lubrication with liquid Vaseline, into the rectum of the mouse (to about 2 cm) and left in position until the stabilization of the temperature (about 10 s; Vigolo et al. 2015). The probe was connected to a digital thermometer (Cole Parmer, model 8402). A cut-off for core body temperature was set at 22 °C (room temperature) as the lowest value reached by animals. The body surface temperature was measured by a Microlife FR 1DZ1 digital infrared digital thermometer (Microlife AG Swiss Corporation, Widnau, Switzerland) placed at 1 cm from the surface of the abdomen of the mouse and left in position until the indication of the temperature on the display of the instrument.

#### Stimulated motor activity assessment

Alteration of stimulated motor activity induced by 5-MeO-MiPT in mice was evaluated through two tests in which the animal is forced to move (accelerod and drag tests; Canazza et al. 2016; Bilel et al. 2020; De-Giorgio et al. 2020). For the accelerod test, mice were placed on a rotating cylinder (Ugo Basile, Milan, Italy) at 5-min intervals. The speed automatically and constantly increased (0–60 rotation/min). Time spent on the moving cylinder was measured with a cut-off of 300 s. The drag test consisted of lifting the mouse by the tail, leaving the front paws on the table, and dragging the animal backward at a constant speed of about 20 cm/s for a distance of 100 cm. The number of steps of each paw was recorded by two different observers. For each animal, five measurement were collected.

#### Startle/prepulse inhibition analysis

As previously reported (Bilel et al. 2020; Corli et al. 2023), mice were tested for acoustic startle reactivity in startle chamber (Ugo Basile apparatus, Milan, Italy) consisting of a sound-attenuated, lighted, and ventilated enclosure holding a transparent non-restrictive Perspex® cage (90 × 45 × 50 mm). Acoustic stimuli were produced by a loudspeaker mounted laterally the holder. Peak and amplitudes of the startle response were detected by a loadcell. At the onset of the startling stimulus, 300-ms readings were recorded and the wave amplitude evoked by the movement of the animal startle response was measured. Acoustic startle test session consisted of startle trials (pulse-alone) and prepulse trials (prepulse + pulse). The pulse-alone trials consisted of a 40-ms 120-dB pulse. Prepulse + pulse trials sequence consisted of a 20-ms acoustic prepulse, a 80-ms delay, followed by a 40-ms 120-dB startle pulse (100-ms onset-onset). Between the trials, there was an average of 15 s (range: from 9 to 21 s). Each startle session began with a 10-min acclimatation period with a 65-dB broadband white noise, continuously present throughout the session. The test session contained 40 trials composed by pulse-alone and prepulse + pulse trials, with three different prepulses of 68-dB, 75-dB, and 85-dB, presented in a pseudorandomized order. Animals were placed in the startle chambers 5 min after treatment and the entire PPI test lasted 20 min. As previously reported, 5-MeO-MiPT (0.01–10 mg/kg) was administered intraperitoneally, and startle/PPI responses were recorded 15 and 120 min after drug injection. The prepulse inhibition (% PPI) was expressed as the percentage decrease in the amplitude of the startle reactivity caused by the presentation of the prepulse.

### Evaluation of cardiorespiratory and blood pressure changes

Cardiorespiratory parameters were monitored in awake and freely moving animals, without using invasive instruments and handling, a collar equipped with a sensor monitored heart rate, breath rate, oxygen saturation (SpO2), and pulse distension with an acquisition frequency of 15 Hz (Bilel et al. 2019; Marchetti et al. 2023). During the procedure, mice freely moved around their cages (30 × 30 × 20 cm), while having no access to food or water and while being monitored by the sensor collar through the software MouseOx Plus (STARR Life Sciences® Corp, Oakmont, PA, USA). To minimize the potential stress of mice during the experiment, a fake collar, similar in design to the one used in the test, was used in the first hour of acclimatation. After that, the collar with the sensor was applied, while the baseline parameters were monitored for 60 min. Then, 5-MeO-MiPT (30 mg/kg) or vehicle was administered, and data were recorded for 5 h.

Systolic and diastolic blood pressure were measured by tail-cuff plethysmography using a BP-2000 blood pressure analysis system (Visitech Systems, Apex, NC, USA; Ossato et al. 2018). Mice were placed in a metal box restraint with the tail passing through the optical sensor and compression cuff before finally being tapped to the platform. A traditional tail-cuff occlude was placed proximally on the animal’s tail, which was then immobilized with tape in a V-shaped block between a light source above and a photoresistor below. Upon inflation, the occlude stopped blood flow through the tail, while upon deflation, the sensor detected the blood flow return. The restraint platform was maintained at a temperature of 37 °C. Before experiments, mice were acclimated to restraint and tail-cuff inflation for 5–7 days. On the test day, 10 measurements were made to collect basal blood pressure. Upon the tenth analysis, the software was paused, and mice were injected with 5-MeO-MiPT (30 mg/kg) or vehicle; animals were then repositioned in the restraints, and 60 measurements were acquired.

### Data and statistical analysis

In sensorimotor response experiments, data are expressed in arbitrary units (visual object response, vibrissae, corneal and pinnae reflex, acoustic response) and percentage of baseline (visual placing response, accelerod, drag tests). Core temperature and body surface temperature values were expressed as the difference between control temperature (before injection) and temperature following drug administration (Δ°C). The amount of PPI was calculated as a percentage score for each prepulse + pulse trial type: % PPI = 100 − {[(startle response for prepulse + pulse trial)/(startle response for pulse-alone trial)] × 100}. Startle magnitude was calculated as the average response to all the pulse-alone trials. All data are shown as mean ± SEM of 8 independent experimental replications. Statistical analysis of the effects of 5-MeO-MiPT at different concentrations over time was performed by two-way ANOVA followed by Bonferroni’s test for multiple comparisons, while statistical analysis of PPI results was carried out with one-way ANOVA followed by the Bonferroni’s test for multiple comparisons and expressed in histograms. Data related to heart rate (heart beats per min; bpm), pulse distention (vessel diameter changes; µm), breath rate (respiratory rate per minute; rrpm), and SpO2 saturation (oxygen blood saturation) were expressed as % changes of basal values. The statistical analysis of the effects of 5-MeO-MiPT (30 mg/kg) was performed by two-way ANOVA followed by Bonferroni’s test for multiple comparisons. Changes in systolic and diastolic blood pressure were expressed as absolute values (mmHg). The effects of 5-MeO-MiPT (30 mg/kg) over time were analysed by a two-way ANOVA followed by Bonferroni’s test for multiple comparisons. All statistical analyses were performed using GraphPad Prism software (GraphPad Prism, San Diego, CA, USA).

## *In silico* ADMET prediction of 5-MeO-MiPT, 5-MeO-DIPT, and DMT

The in silico ADMET prediction of 5-MeO-MiPT, 5-MeO-DIPT, and DMT (chemical structures, Fig. 1) profiles was conducted through Simulations Plus ADMET Predictor® version 10.4 (× 64) on a Windows 11 operating system (ADMET Predictor®, Simulations-Plus Inc). The program predicts the physicochemical, pharmacokinetics, and toxicity properties based on the molecular structures of compounds. It uses artificial neural network ensemble (ANNE) models, which were trained to ensemble with data sets that share the same “architecture” (i.e. same inputs and number of neurons) from well-defined drugs, using the 2D structure and the atomic descriptors for data selection. The criteria used to set the ADMET scores are illustrated in Table 1.

**Table 1:** Overview of ADMET parameters with their recommended ranges

Parameter	Recommended range	Comments
ADMET_Risk^a^	< 7	Includes components of all risk models as well as fraction unbound to plasma and volume of distribution
Absn_Risk^b^	< 4	Considers size, rotational bonds, hydrogen bonding capacity, polar surface area, permeability, lipophilicity, solubility
TOX_Risk^c^	< 2	Consists of hERG^l^, acute toxicity in rats, carcinogenicity in chronic rat/mouse studies, hepatotoxicity, mutation
CYP_Risk^d^	< 2	Includes inhibition of CYPs^m^ 1A2, 2C19, 2C9, 2D6, and 3A4, excessive clearance, and inhibition of midazolam or testosterone
Peff^e^ [cm/s × 10^4^]	≥ 0.5	Jejunal permeability S + Peff < [0.40, 0.60 cm/s × 10^4^]
MDCK^f^ [cm/s × 10^7^]	≥ 30	S + MDCK > 30 cm/s × 10^7^ indicates high permeability
Fup^g^ [%]	> 10%	hum_fup% and mou_fup% < 10% indicates extensive plasma binding
RBP^h^	< 1.0	hum_RBP and mou_RBP > 1 indicates a partitioning to erythrocytes
Vd^i^ [L/kg]	≤ 3.7	Vd > 3.7 indicates high distribution at steady state

^a^Absorption, distribution, metabolism, excretion, and toxicity risk; ^b^absorption risk; ^c^toxicity risk; ^d^cytochrome risk; ^e^jejunal permeability; ^f^Madin-Darby canine kidney; ^g^percent unbound to blood plasma proteins; ^h^blood to plasma ratio; ^i^volume of distribution; ^l^human voltage-sensitive K + channel inhibition; ^m^cytochromes P450

## Results

### Case report

In July 2022, a 23-year-old male, transported via 112 emergency ambulance, was admitted to the Emergency Department (ED) of a Northern Italy Hospital manifesting tachycardia, catatonia, slowdown, drowsiness, and muteness. The anamnestic evaluation revealed a medical history characterized by a pre-existing depressive disorder, medicated with sertraline and alprazolam. The mother reported that, together with the ingestion of alprazolam, the son had smoked and sniffed an unknown substance, purchased via an internet-order. Therefore, the patient was temporarily diagnosed as suffering from a toxidrome with an unknown substance. His temperature was 36.0 °C, pulse rate 107 bpm, breath rate 19 breaths/min, SpO_2_ 99%, and blood pressure 150/90 mmHg. Standard laboratory investigations assessed borderline haematochemical parameters. The patient’s treatment consisted of a supportive care and symptom management, conducted until the complete remission of symptomatology. The patient was discharged home after 24 h, without any known sequel. During hospitalization, blood and urine samples were collected and then processed by high-performance liquid chromatography-tandem mass spectrometry (LC–MS/MS) and gas chromatography/mass spectrometry (GC/MS) for the toxicological confirmation analyses, also with the support of electronic libraries. The analytical results indicated the presence of 5-MeO-MiPT, 4F-MPH, and DCK. The same samples tested negative for other substances (Table 2).

**Table 2:** Summary of the non-fatal human intoxication case with 5-MeO-MiPT reported by Pavia Poison Control Centre—National Toxicology Information Centre

Eds admission	Age (year), sex, type	Anamnestic data	Symptoms and clinical presentation	Management/therapy	Blood/urine analyses: analytical findings*	Hospital stay and outcome
July 2022; Northern Italy Hospital	23, male, non-fatal intoxication	Pre-existing depressive disorder (pharmacological therapy: sertraline and alprazolam)	- Tachycardia - Catatonia - Slowdown - Drowsiness - Muteness	Symptomatic treatment	5-MeO-MiPT; 4F-MPH; DCK **#**	24 h Discharged home

*4-F-MPH*, 4-fluoromethylphenidate; *DCK*, deschloroketamine

*GC/MS and LC–MS/MS methods

**#**Tested negative for the following substances: butylone, eutylone, ephylon, mephedrone, clephedrone, methylcathinone, dimethylcathinone, N-ethylpentedrone, MDPEP, dimethylmethcathinone, buphedrone, ethcathinone, 4-fluoromethcathinone, pentedrone, methedrone, mexedrone, ethylone, pentylone, naphyrone, methylone, α-PHP, 3,4-MDPHP, α-PVP, MDPV, betaK-2CB, PMA, PMMA, 4-fluoroamphetamine, 4-MTA, ketamine, 2-fluoroketamine, OH-PCP, OH-PCE, MeO-PCE, MeO-PCP, OXO-PCE, levamisole, scopolamine, atropine, (aminopropyl)benzofurans, 5-EAPB, 5/6-MAPB, 4-OH-DET, 5-MeO-DALT, 5-Meo-DMT, methylphenidate, ethylphenidate, phencyclidine, methoxetamine, methoxphenidine, diphenidine, dimethyltryptamine, 2C-I, 2C-T7, 2C-B, 2C-T2, 2C-E, DOB, 25B-NBOMe, 25C-NBOMe, 25I-NBOMe, 25H-NBOMe, 25T7-NBOMe, 25D-NBOMe, and 25E-NBOMe

### Behavioural tests

#### Evaluation of visual response (object and placing response tests)

**Fig. 2:**
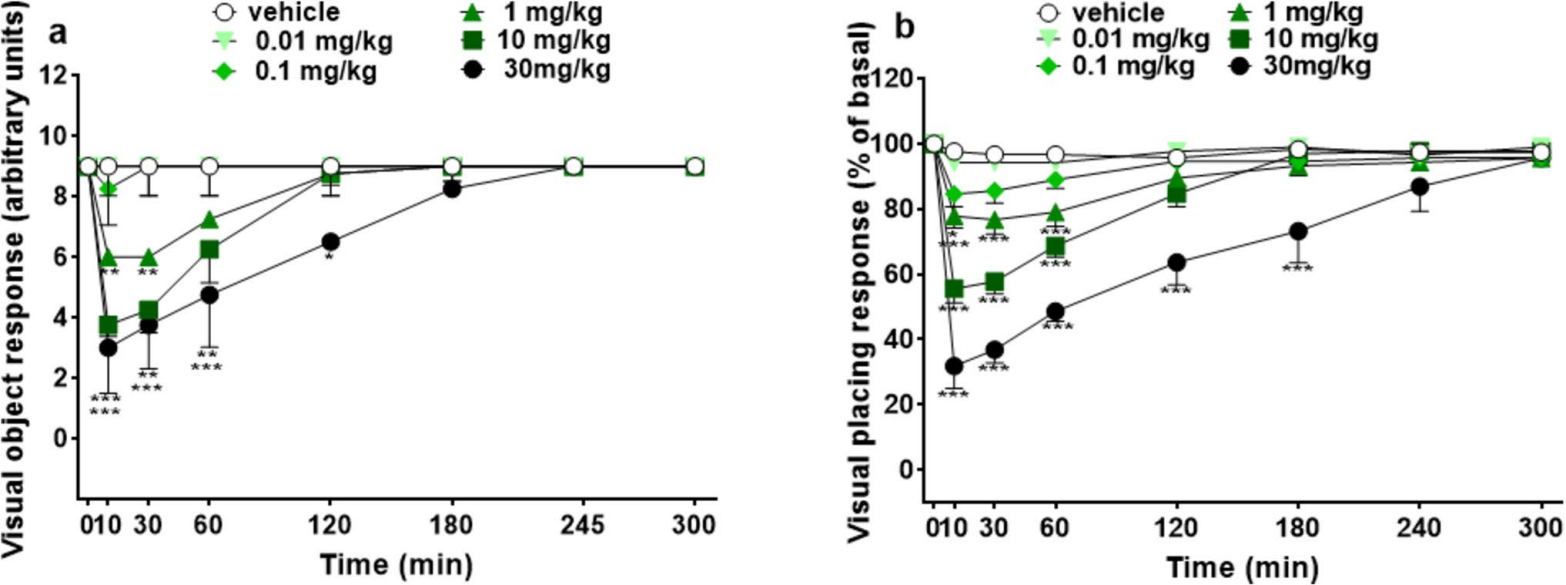
Effects of 5-MeO-MiPT (0.01, 0.1, 1, 10, and 30 mg/kg; i.p.) on visual object (**a**) and placing (**b**) responses in mice. Data are expressed as mean ± SEM (*n* = 8/group). Statistical analysis was performed by two-way ANOVA followed by Bonferroni’s test for multiple comparisons for the dose–response curve of each compound at different time points (*p < 0.05, **p < 0.01, ***p < 0.001 versus vehicle)

Visual object (both frontal and lateral; Fig. 2a) and placing (Fig. 2b) responses did not change in vehicle-treated mice during 5 h of observation. Systemic administration of low doses (0.01–0.1 mg/kg i.p.) of 5-MeO-MiPT did not affect the visual object response (Fig. 1a). The dose of 1 mg/kg reduced the visual object response of mice ~ 33% compared to basal values during the first 30 min of the experiment [significant effect of treatment (*F*_5,336_ = 20.65, *p* < 0.0001), time (*F*_7,336_ = 17.35, *p* < 0.0001), and time × treatment interaction (*F*_35,336_ = 3.700, *p* < 0.0001)]. Ten and 30 mg/kg (i.p.; Fig. 2a) more deeply decreased the visual reflexes of animals in a transient manner (~ 55% at 10 min). In particular, after 120 min of treatment with 10 mg/kg 5-MeO-MiPT the response was equal to basal values, while for the 30 mg/kg was persistent. Systemic administration of 5-MeO-MiPT dose-dependently (Fig. 2b) reduced the visual placing response in mice [significant effect of treatment (*F*_5,336_ = 97.11, *p* < 0.0001), time (*F*_7,336_ = 59.28, *p* < 0.0001), and time × treatment interaction (*F*_35,336_ = 9.836, *p* < 0.0001)]. All the doses transiently induced an inhibition of the response, except the lowest one (0.01 mg/kg) that did not affect the visual placing response. The 0.1 mg/kg of 5-MeO-MiPT had a mild effect only during the first 5 min, while the highest dose (30 mg/kg) impaired the visual reflexes of mice to ~ 80% compared to the basal values during the first 5 min (*p* < 0.001) and the effect persisted up to 180 min (~ 30%) of observation.

#### Evaluation of overall tactile and acoustic responses

**Fig. 3:**
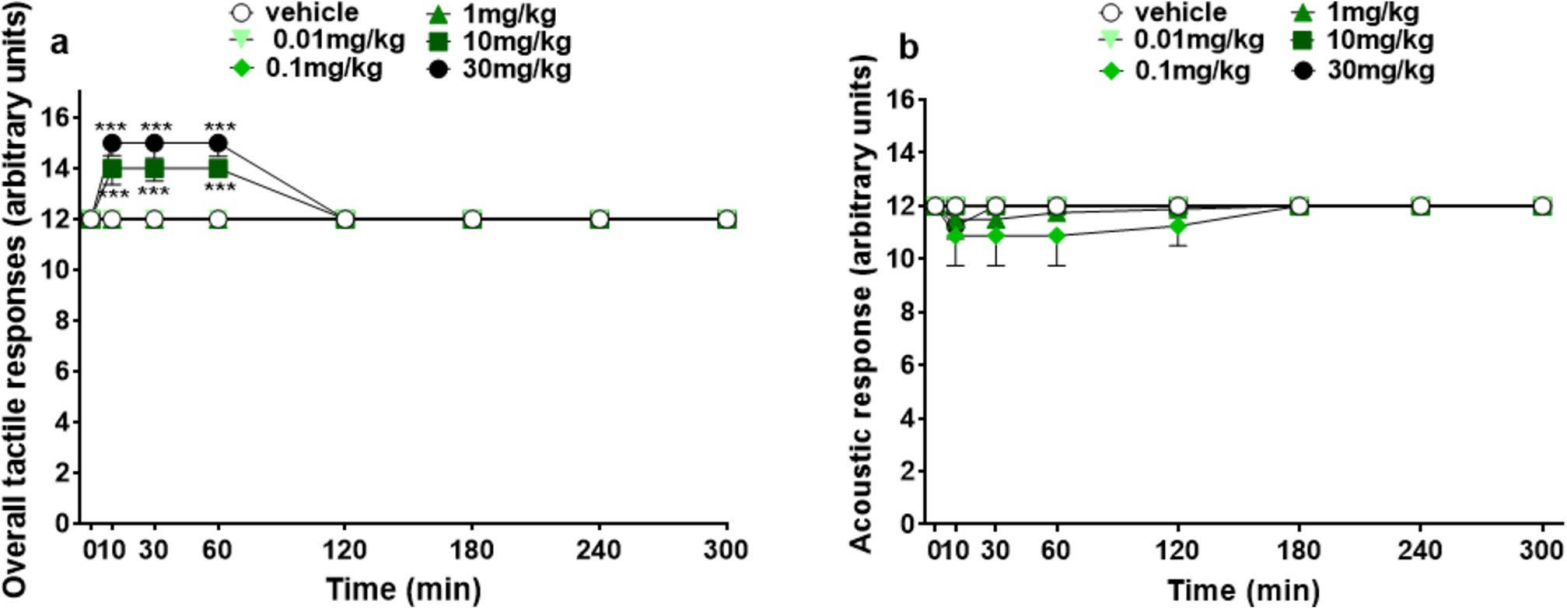
Effects of 5-MeO-MiPT (0.01, 0.1, 1, 10, and 30 mg/kg; i.p.) on overall tactile (**a**) and acoustic (**b**) responses in mice. Data are expressed as mean ± SEM (*n* = 8/group). Statistical analysis was performed by two-way ANOVA followed by Bonferroni’s test for multiple comparisons for the dose–response curve of each compound at different time points (***p < 0.001 versus vehicle)

Overall tactile and acoustic responses did not change in vehicle-treated mice during 5 h of observation (Fig. 3a, b). Systemic administration of 5-MeO-MiPT (0.01–1 mg/kg) did not affect the tactile responses of mice. The injection of 10–30 mg/kg induced a significant enhancement of the reflex to tactile stimulation during the first 60 min of the experiment (Fig. 3a) and the effect disappeared after 120 min of treatment [significant effect of treatment (*F*_5,336_ = 58.99, *p* < 0.0001), time (*F*_7,336_ = 33.12, *p* < 0.0001), and time × treatment interaction (*F*_35,336_ = 14.04, *p* < 0.0001)].

Conversely, the systemic administration of 5-MeO-MiPT (0.01–30 mg/kg) did not significantly affect the acoustic response of mice (Fig. 3b). Two-way ANOVA detected a significant effect of treatment [*F*_5,336_ = 3.160, *p* = 0.0084], time [*F*_7,336_ = 1.410, *p* = 0.2004], and time × treatment interaction [*F*_35,336_ = 0.5742, *p* = 0.9760].

#### Evaluation of core temperature and body surface temperature

**Fig. 4:**
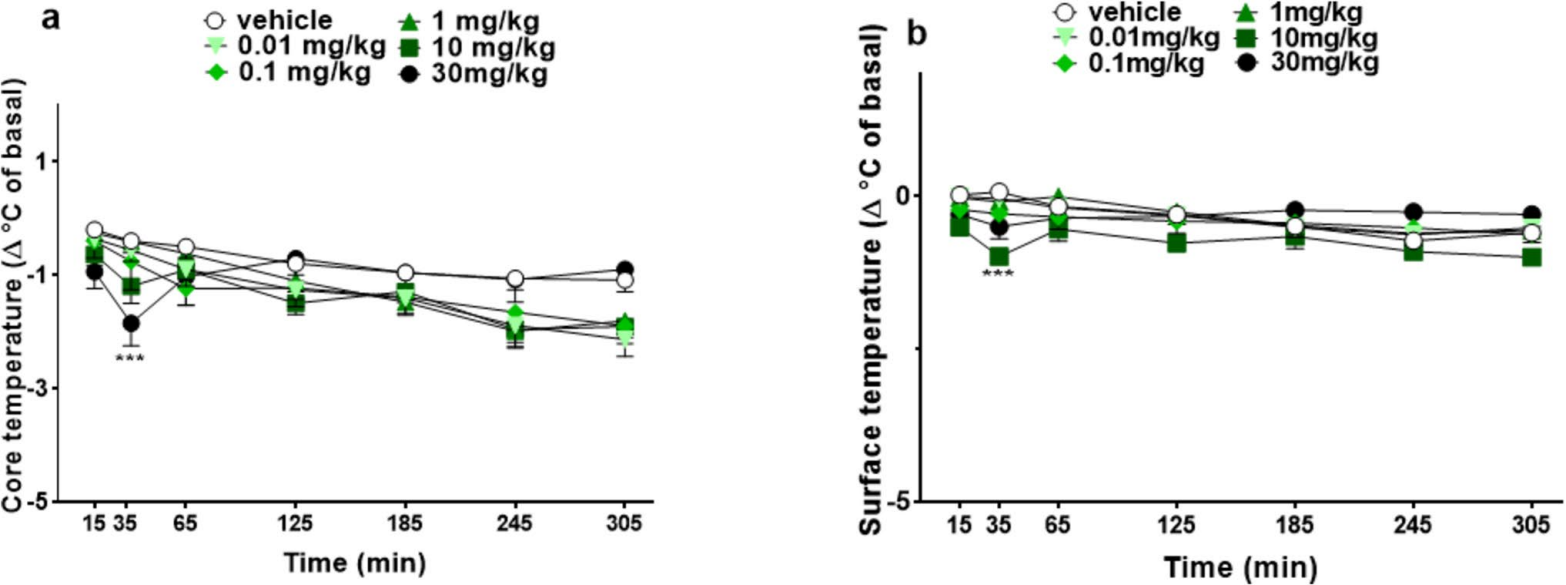
Effects of 5-MeO-MiPT (0.01, 0.1, 1, 10, and 30 mg/kg; i.p.) on core body temperature (**a**) and body surface temperature (**b**) in mice. Data are expressed as mean ± SEM (*n* = 8/group). Statistical analysis was performed by two-way ANOVA followed by Bonferroni’s test for multiple comparisons for the dose–response curve of each compound at different time points (***p < 0.001 versus vehicle)

Core temperature and body surface temperature of vehicle-treated animals remained constant during 5 h of observation (Fig. 4a, b). Only the highest dose of 5-MeO-MiPT (30 mg/kg i.p.) induced a hypothermic effect (Fig. 4a) at 35 min from the administration [significant effect of treatment (*F*_5,294_ = 4.486, *p* = 0.0006), time (*F*_6,294_ = 14.38, *p* < 0.0001), and time × treatment interaction (*F*_30,294_ = 1.625, *p* = 0.0238)]. On body surface temperature (Fig. 4b), two-way ANOVA revealed a significant decrease at the dose of 10 mg/kg at 35 min after treatment [significant effect of treatment (*F*_5,294_ = 11.85, *p* < 0.0001), time (*F*_6,294_ = 8.400, *p* < 0.0001), and time × treatment interaction (*F*_30,294_ = 1.262, *p* = 1.2692)].

#### Stimulated motor activity assessment

**Fig. 5:**
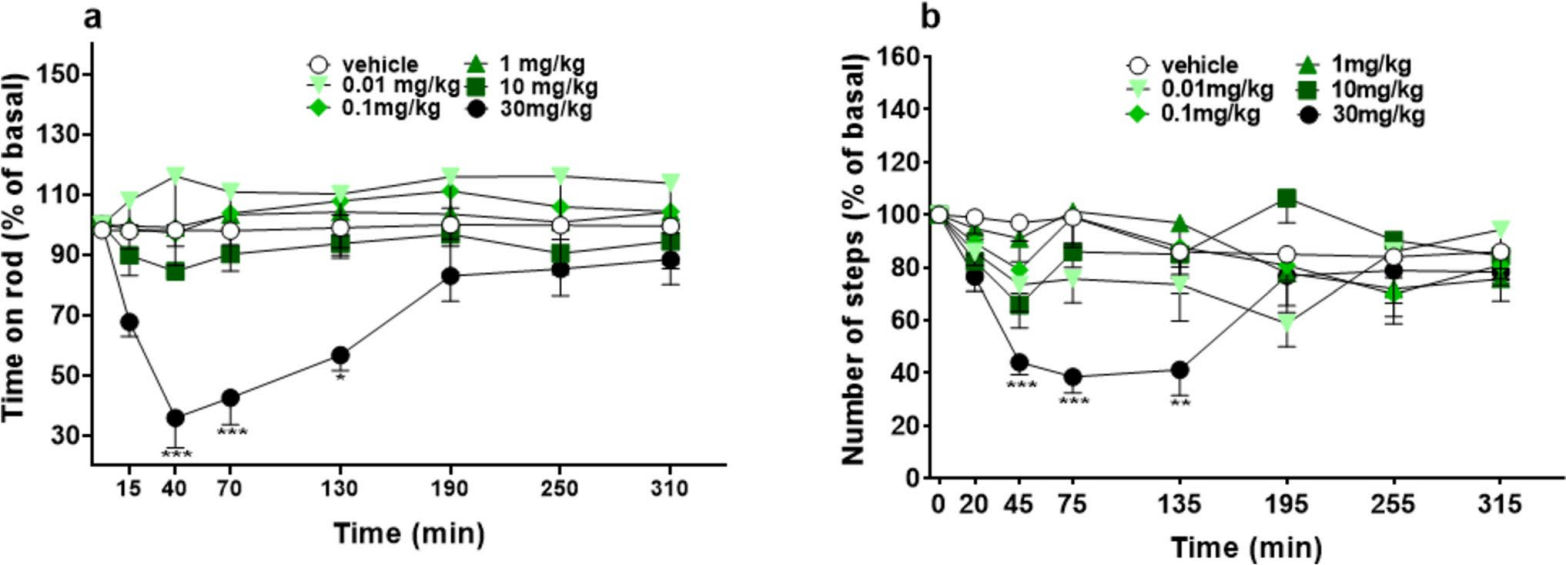
Effects of 5-MeO-MiPT (0.01, 0.1, 1, 10, and 30 mg/kg; i.p.) on time on rod (**a**) and number of steps (**b**) in mice. Data are expressed as mean ± SEM (*n* = 8/group). Statistical analysis was performed by two-way ANOVA followed by Bonferroni’s test for multiple comparisons for the dose–response curve of each compound at different time points (*p < 0.05, **p < 0.01, ***p < 0.001 versus vehicle)

Vehicle administration did not change motor activity in both accelerod and drag tests over the 5 h observation (Fig. 5a, b) in mice. The motor performance was not significantly affected in the groups of mice treated with the doses of 0.01, 0.1, 1, and 10 mg/kg of 5-MeO-MIPT (Fig. 4a). The highest dose induced a strong inhibition of the motor performance in the accelerod test (~ 70% of the basal), the effect appeared at 40 min after the administration, at 130 min there was still a mild effect, and in the last 2 h of the experiment the performance returned to basal values. Two-way ANOVA detected a significant effect of treatment [*F*_5,336_ = 20.16, *p* < 0.0001], time [*F*_7,336_ = 1.745, *p* = 0.0978], and time × treatment interaction [*F*_35,336_ = 1.163, *p* = 0.2480]. Similarly, the systemic administration of 5-MeO-MiPT (0.01–30 mg/kg) did not induce any effect on drag test, except with the highest one (Fig. 5b). In fact, the injection of 30 mg/kg induced ~ 60% of reduction of the number of steps performed with the front paws of mice after 45 min from the injection and the effect persisted up to 135 min of observation [significant effect of treatment (*F*_5,336_ = 7.673, *p* < 0.0001), time (*F*_7,336_ = 4.116, *p* = 0.0002), and time × treatment interaction (*F*_35,336_ = 1.828, *p* = 0.0038)].

#### Startle/prepulse inhibition (PPI) analysis

**Fig. 6:**
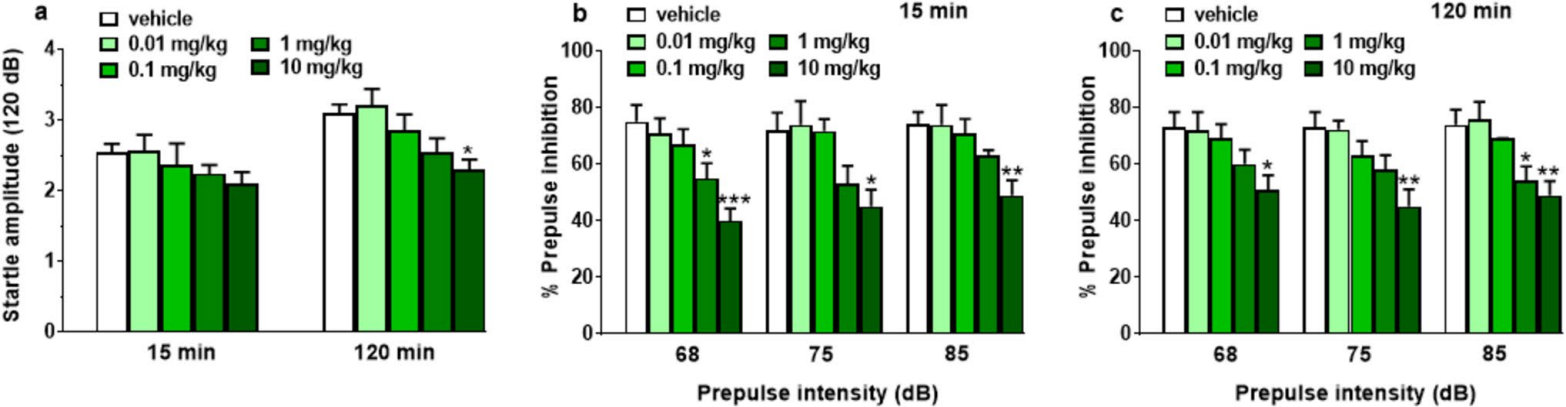
Effects of 5-MeO-MiPT (0.01, 0.1, 1, 10, and 30 mg/kg; i.p.) on startle amplitude (**a**) and prepulse inhibition (PPI; **b**, **c**) in mice. Effects on PPI are shown for the three prepulse intensities (68, 75, 85 dB) 15 min (**b**) and 120 min (**c**) after treatment. Startle amplitude was expressed in absolute values (in dB) and the values represent the mean ± SEM (*n* = 8/group). PPI was expressed as the percentage decrease in the amplitude of the startle reactivity caused by presentation of the prepulse (% PPI; see “Materials and methods” section), values represent mean ± SEM (*n* = 8/group). Statistical analysis was performed by one-way ANOVA followed by Bonferroni’s test for multiple comparisons (*p < 0.05, **p < 0.01, and ***p < 0.001 vs. vehicle)

Startle and PPI response did not change after the vehicle injection in mice (Fig. 6a–c). The systemic administration of 5-MeO-MiPT reduced the acoustic startle amplitude only at the dose of 10 mg/kg (Fig. 6a) at 120 min of analysis [significant effect of treatment (*F*_4,35_ = 3.862, *p* = 0.0106)]. In addition, PPI was also impaired after 1 mg/kg of 5-MeO-MiPT treatment and one-way ANOVA detected a significant effect at 15 min (Fig. 6b) at 68 dB [significant effect of treatment *F*_4,35_ = 7.340, *p* = 0.0002] and at 120 min (Fig. 6c) at 85 dB [significant effect of treatment *F*_4,35_ = 6.431, *p* = 0.0005].

The injection of 10 mg/kg of 5-MeO-MiPT caused an impairment of the prepulse inhibition at 15 min (Fig. 6b) at 68 dB [significant effect of treatment (*F*_4,35_ = 7.340, *p* = 0.0002)], 75 dB [significant effect of treatment (*F*_4,35_ = 4.362, *p* = 0.0058)], and 85 dB [significant effect of treatment (*F*_4,35_ = 4.631, *p* = 0.0042)]. The effect persisted after 120 min of analysis (Fig. 6c); one-way ANOVA detected a reduction of the PPI at 68 dB [significant effect of treatment (*F*_4,35_ = 2.953, *p* = 0.0334)], 75 dB [significant effect of treatment (*F*_4,35_ = 5.258, *p* = 0.0020)], and 85 dB [significant effect of treatment (*F*_4,35_ = 6.431, *p* = 0.0005)].

### Cardiorespiratory analysis

**Fig. 7:**
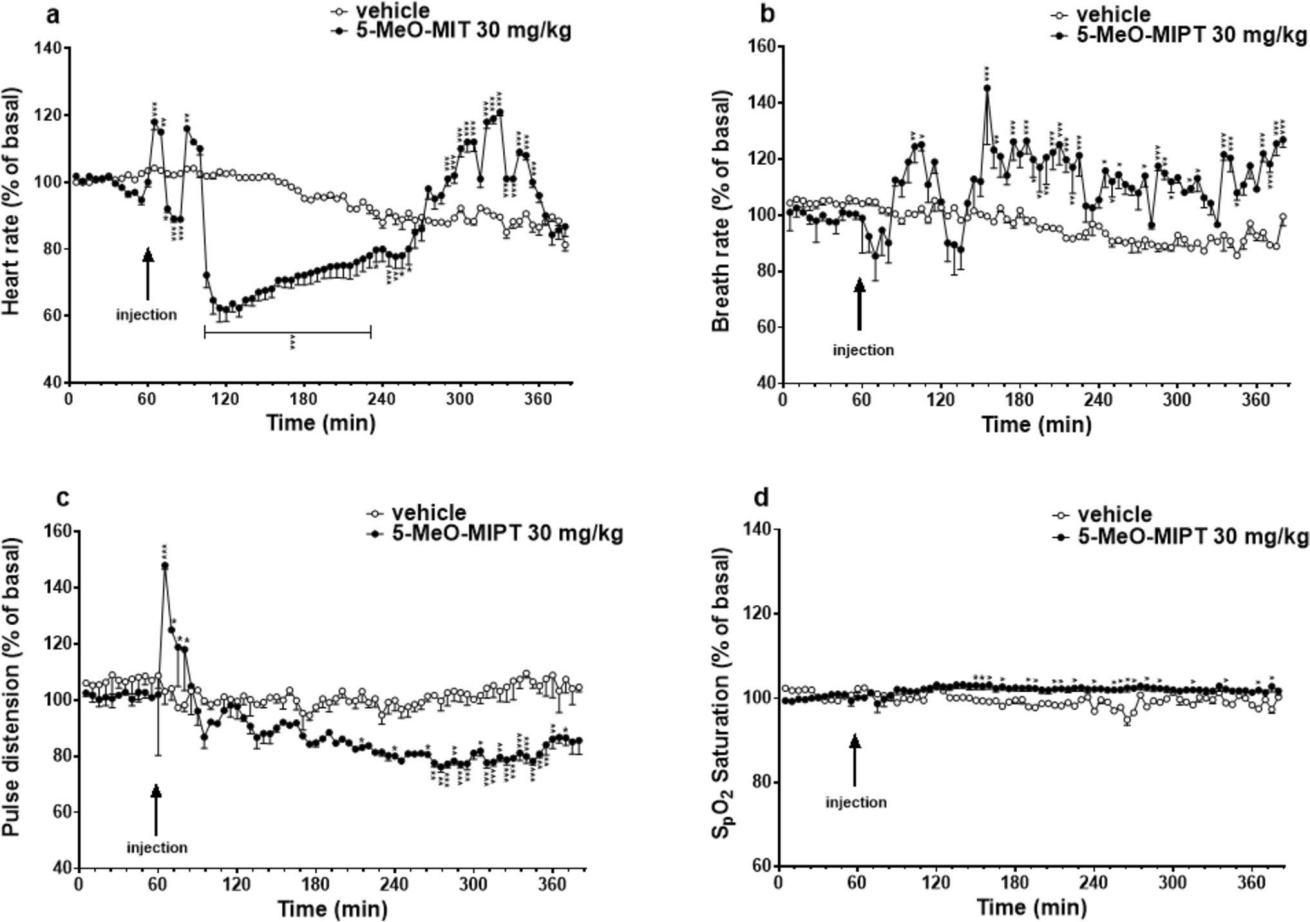
Effects of 5-MeO-MiPT (30 mg/kg; i.p.) on heart rate (**a**), breath rate (**b**), pulse distension (**c**), and SpO2 saturation (**d**) in mice. Data are expressed as percentage of basal values in the form mean ± SEM (*n* = 6/group). Statistical analysis was performed by two-way ANOVA, followed by Bonferroni’s test for multiple comparisons (*p < 0.05, **p < 0.01, *** *p* < 0.001 versus vehicle)

Since the most pronounced effects were observed after the systemic administration of the highest dose tested of 5-MeO-MiPT (30 mg/kg), we decided to investigate its possible adverse effects on cardiorespiratory system in mice, simulating a heavy intoxication. Basal heart rate (679 ± 14 bpm), breath rate (202 ± 2.42 rrpm), pulse distension (vessel diameter, 344 ± 16.83 µm), and SpO2 (92.22 ± 0.37 SpO2) did not change in animals treated with the vehicle (Fig. 7a–d). The systemic administration of 5-MeO-MiPT evoked a triphasic effect in heart rate in mice (Fig. 7a). After an initial enhancement right after the injection, a significant reduction in heart rate (between 40 and 20% comparing to basal values) was observed and the bradycardic effect lasted about 2 and a half hours of analysis. Then, from 255 min after the treatment the heart rate increased again, the maximal increase (~ 20%) was recorded at 330 min of the experiment. Two-way ANOVA revealed a significant effect of treatment [*F*_1,760_ = 412.8, *p* < 0.0001], time [*F*_75,760_ = 34.66, *p* < 0.0001], and time × treatment interaction [*F*_75,760_ = 43.93, *p* < 0.0001]. Simultaneously, the breath rate raised in the first hour of injection (Fig. 7b). The effect disappeared at 120 min after injection and manifested again at 140 min of analysis. The maximal effect (~ 150%) was registered at 155 min and the tachypnoea persisted up to the end of the experiment [significant effect of treatment (*F*_1,760_ = 366.2, *p* < 0.0001), time (*F*_75,760_ = 3.180, *p* < 0.0001), and time × treatment interaction (*F*_75,760_ = 5.380, *p* < 0.0001)]. The pulse distention (Fig. 7c) also immediately increased (~ 150% of basal values) after the 5-MeO-MiPT injection and then significantly decreased during the last 3 h of the experiment [significant effect of treatment (*F*_1,760_ = 374.9, *p* < 0.0001), time (*F*_75,760_ = 5.744, *p* < 0.0001), and time × treatment interaction (*F*_75,760_ = 4.768, *p* < 0.0001)]. At the same time, administration of 5-MeO-MIPT did not cause pronounced variation in SpO2 (Fig. 7d), two-way ANOVA detected a mild but statistically significant increase of Sp02 in comparison to basal values from 90 min after the treatment, and it remained constant during the following hours of the experiment [significant effect of treatment (*F*_1,760_ = 386.6, *p* < 0.0001), time (*F*_75,760_ = 2.259, *p* < 0.0001), and time × treatment interaction (*F*_75,760_ = 5.371, *p* < 0.0001)].

**Fig. 8:**
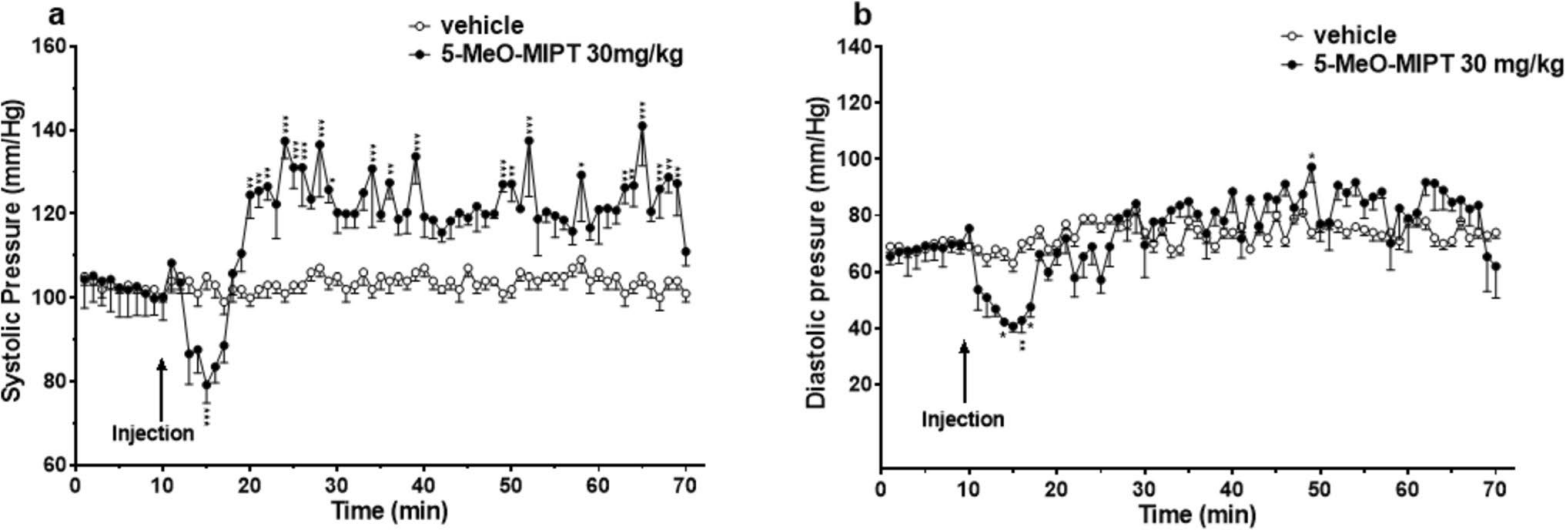
Effects of 5-MeO-MiPT (30 mg/kg; i.p.) on systolic (**a**) and diastolic (**b**) blood pressure. Data are expressed as absolute values (mm/Hg) in the form mean ± SEM (*n* = 5/group). Statistical analysis was performed by two-way ANOVA, followed by Bonferroni’s test for multiple comparisons (*p < 0.05, **p < 0.01, ***p < 0.001 versus vehicle)

Basal systolic (103 ± 2 mmHg) and diastolic (73 ± 2 mmHg) pressure did not change in the group of mice treated with the vehicle (Fig. 8a, b). The systemic administration of 5-MeO-MIPT induced an immediate decrease of the systolic pressure (~ 80 mmHg; Fig. 8a) during the first 10 min after the injection followed by an increase that lasted up to the end of registration. Two-way ANOVA detected significant effect of treatment [*F*_1,630_ = 376.9, *p* < 0.0001], time [*F*_69,360_ = 5.641, *p* < 0.0001], and time × treatment interaction [*F*_69,630_ = 4.870, *p* < 0.0001]. Diastolic pressure also decreased during the first minutes after the injection (~ 40 mmHg), then it raised after 10 min (Fig. 8b). Then, ANOVA analysis detected a statistically mild significant increase of diastolic pressure only at 49 min of the experiment [significant effect of treatment (*F*_1,560_ = 3.239, *p* = 0.0724), time (*F*_69, 560_ = 5.342, *p* < 0.0001), and time × treatment interaction (*F*_69,560_ = 3.094, *p* < 0.0001)].

### ADMET prediction of 5-MeO-MiPT, 5-MeO-DIPT, and DMT

ADMET Predictor® calculated the risk scores for 5-MeO-MiPT, 5-MeO-DIPT, and DMT (Table 3). The ADMET risk of 5-MeO-MiPT, its analogue 5-MeO-DIPT, and the indole-alkaloid DMT were below the threshold of 7; however, ADMET predicted potential risk codes related to the volume of distribution (Vd) for 5-MeO-DIPT and DMT. The predictor calculated no absorption risk for the three compounds, and no toxicity risk for 5-MeO-MiPT and 5-MeO-DIPT. The predicted toxicity risk for DMT was under the threshold of 2, with a risk code related to acute rat toxicity (Table 3). ADMET predicted no cytochrome risk for DMT, whereas the scores of 5-MeO-MiPT and 5-MeO-DIPT were below the cytochrome risk threshold (< 2). Cytochrome risk code of high clearance by cytochrome P450 1A2 (CYP1A2) was predicted for both compounds, while risk code of high clearance by cytochrome P450 2C9 (CYP2C9) was predicted only for 5-MeO-MiPT. For DMT, a potential mutation risk was also predicted (yellow flag plot, Table 3); however, this risk is not considered in our study. As shown in Table 4, the three compounds were not predicted to induce elevated levels of hepatic enzymes in human serum, except for DMT which is predicted to induce elevated levels of serum aspartic acid transaminase (Ser_AST).

**Table 3:**
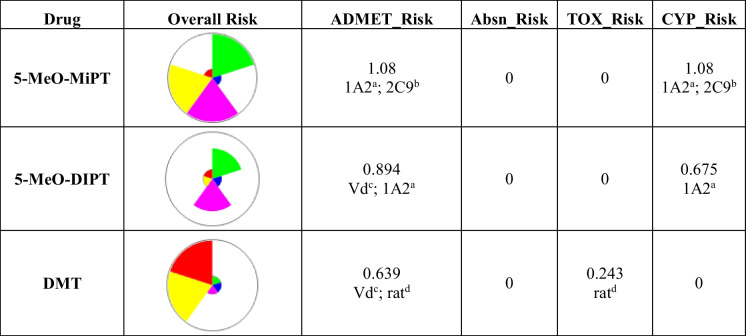
Risk scores of 5-MeO-MiPT, 5-MeO-DIPT, and DMT by ADMET Predictor®. The wedge colours in the star plots correspond to the following: green for ADMET_Risk, blue for Absn_Risk, red for TOX_Risk, purple for CYP_Risk, and yellow for MUT_Risk

^a^High clearance by cytochrome P450 1A2 (CYP1A2); ^b^high clearance by cytochrome P450 2C9 (CYP2C9); ^c^volume of distribution; ^d^acute rat toxicity

**Table 4:** Predicted effects of 5-MeO-MiPT, 5-MeO-DIPT, and DMT and their percentage of accuracy (%) by ADMET Predictor® on hepatic enzyme levels in human serum

Drug	Ser_AlkPhos^a^	Ser_GGT^b^	Ser_LDH^c^	Ser_AST^d^	Ser_ALT^e^
5-MeO-MiPT	Normal (92%)	Normal (97%)	Normal (94%)	Normal (57%)	Normal (86%)
5-MeO-DIPT	Normal (92%)	Normal (97%)	Normal (94%)	Normal (91%)	Normal (94%)
DMT	Normal (83%)	Normal (97%)	Normal (62%)	Elevated (96%)	Normal (94%)

^a^Serum alkaline phosphatase; ^b^serum gamma-glutamyl transferase; ^c^serum lactate dehydrogenase; ^d^serum aspartic acid transaminase, ^e^serum alanine transaminase

Table 5 describes the absorption profile of 5-MeO-MiPT, 5-MeO-DIPT, and DMT. The predictor calculated a high jejunal permeability (S + Peff) and a very high kidney permeability predicted in Madin-Darby canine kidney cells (MDCK) for the three compounds. They were all predicted to have a high blood brain barrier (BBB) penetration (99%). None of the compounds was predicted to be substrate and/or inhibitor of P-glycoprotein (P-gp) and substrate of the organic anion transporter 1 (OAT1) and organic anion transporter 3 (OAT3).

**Table 5:** Predicted permeability and transporting properties of 5-MeO-MiPT, 5-MeO-DIPT, and DMT and their percentage of accuracy (%) by ADMET Predictor®

Drug	S + Peff^a^ [cm/s × 10^4^]	S + MDCK^b^ [cm/s × 10^7^]	BBB^c^ penetration	Pgp_Sub^d^	Pgp_Inh^e^	OAT1_Sub^f^	OAT3_Sub^g^
5-MeO-MiPT	2.986	715.788	High (99%)	No (80%)	No (93%)	No (92%)	No (86%)
5-MeO-DIPT	2.731	650.151	High (99%)	No (57%)	No (93%)	No (92%)	No (96%)
DMT	3.862	766.624	High (99%)	No (80%)	No (93%)	No (82%)	No (63%)

^a^Jejunal permeability; ^b^Madin-Darby canine kidney; ^c^blood brain barrier; ^d^P-glycoprotein substrate; ^e^P-glycoprotein inhibitor; ^f^organic anion transporting 1 substrate; ^g^organic anion transporting 3 substrate

Some pharmacokinetic properties were also predicted (Table 6) for the three compounds. The predicted percent unbound to blood plasma proteins in human (hum_fup%) of the three drugs were similar to the values predicted for mice (mou_fup%). The calculated hum_fup% of the compounds were all over 30%. DMT was predicted to have a blood to plasma ratio in human (hum_RBP) slightly higher than 5-MeO-MiPT and 5-MeO-DIPT, which showed values lower than 1. Concerning the predicted mouse blood to plasma ratio (mou_RBP), the values of the three drugs were above 1.

**Table 6:** Predicted pharmacokinetic properties of 5-MeO-MiPT, 5-MeO-DIPT, and DMT by ADMET Predictor®

Drug	hum_fup^a^ [%]	mou_fup^b^ [%]	hum_RBP^c^	mou_RBP^d^	Vd^e^ [L/kg]	BSEP_IC50^f^ [µM]	S + CL_Mech^g^	ECCS_Class^h^
5-MeO-MiPT	45.506	31.561	0.92	1.071	3.743	85.931	Metabolism	Class_2
5-MeO-DIPT	31.628	32.452	0.883	1.052	4.219	73.409	Metabolism	Class_2
DMT	55.216	38.284	1.051	1.116	4.396	77.992	Metabolism	Class_2

^a^Percent unbound to blood plasma proteins in human; ^b^percent unbound to blood plasma proteins in mouse; ^c^blood to plasma ratio in human; ^d^blood to plasma ratio in mouse; ^e^volume of distribution; ^f^bile salt export pump IC50; ^g^predicts clearance mechanism as primarily metabolism, renal or hepatic uptake; ^h^Extended Clearance Classification System (based on Varma et al. 2015 publication)

The predicted volumes of distribution (Vd) of the three compounds were high, indicating that they are widely distributed in tissues after administration. The predicted values were higher for 5-MeO-DIPT and DMT than 5-MeO-MiPT, with a resulting potential ADME risk described in Table 3 for the former two compounds.

Regarding the inhibition of the bile salt export pump (BSEP), values of IC50 below the threshold of 60 μM indicate an inhibition of the transporter. The three drugs are not predicted to inhibit BSEP, since the IC50 values exceeded the threshold. The primary clearance mechanism predicted for 5-MeO-MiPT, 5-MeO-DIPT, and DMT was metabolism; in fact, the three compounds are classified as class_2 according to Extended Clearance Classification System (ECCS).

**Fig. 9:**
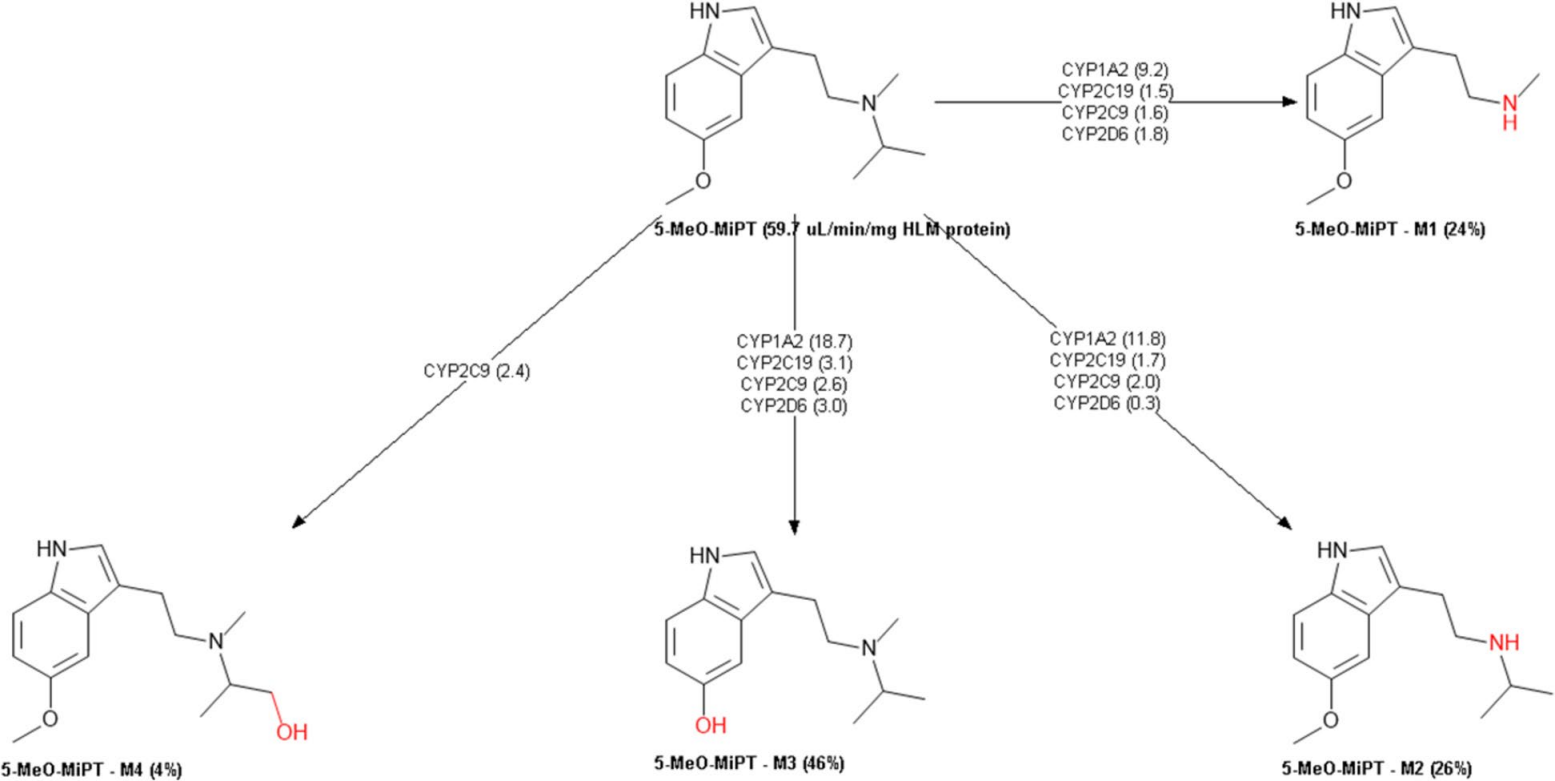
Predicted metabolism of 5-MeO-MiPT by ADMET Predictor®

**Fig. 10:**
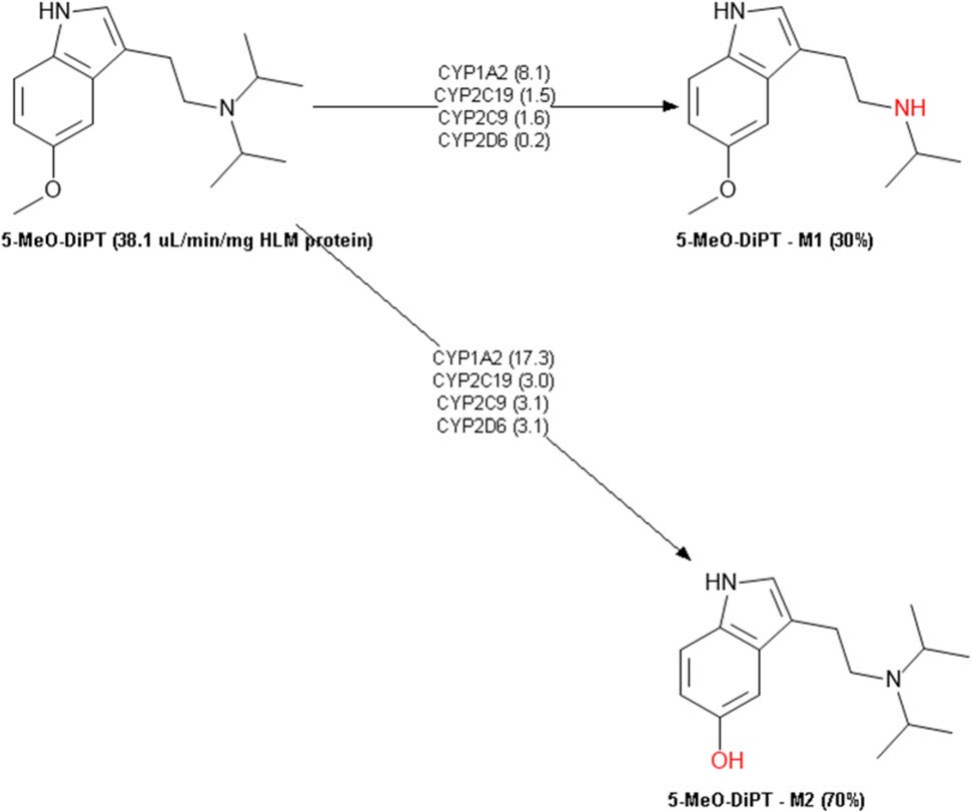
Predicted metabolism of 5-MeO-DIPT by ADMET Predictor®

**Fig. 11:**
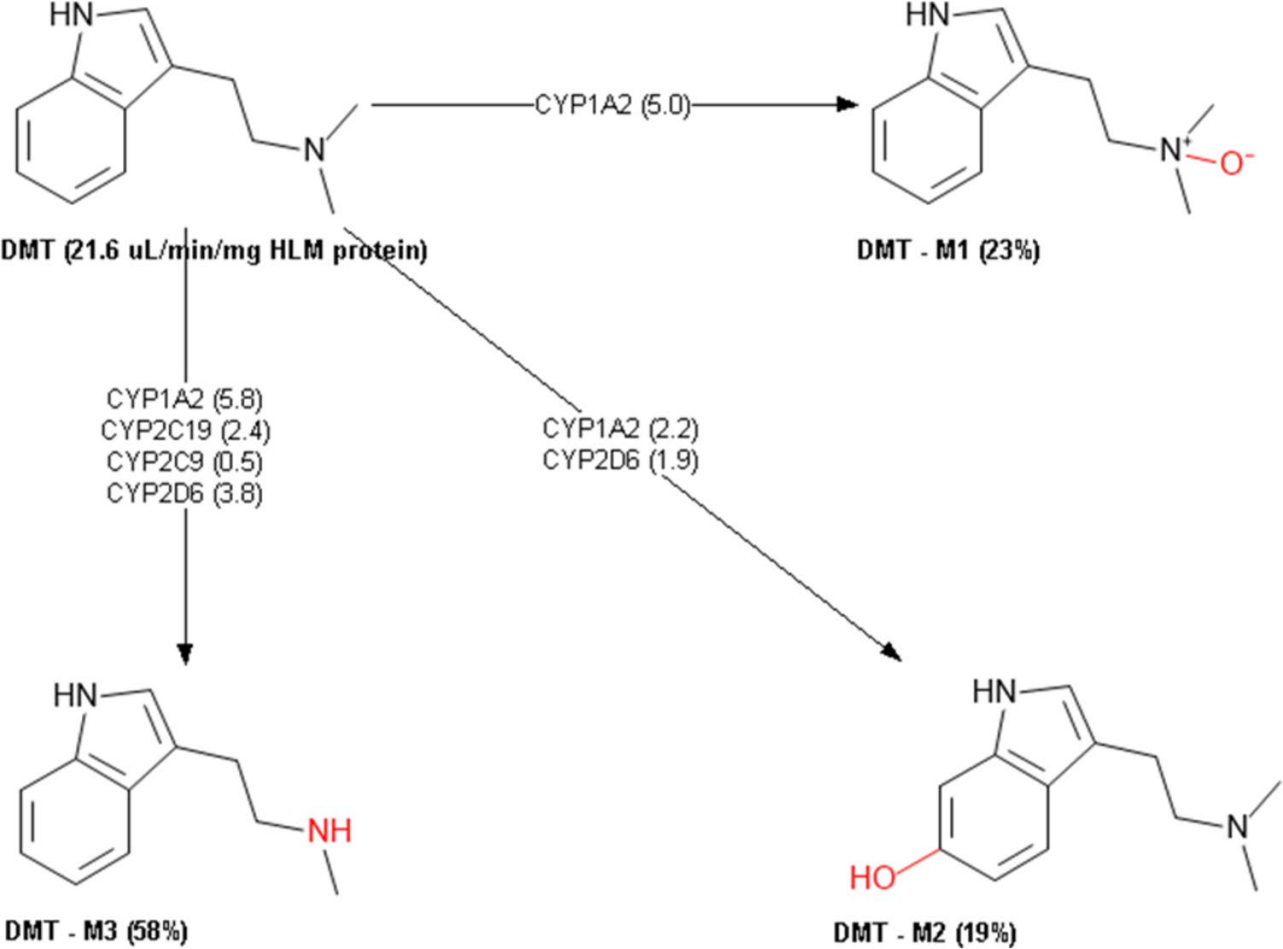
Predicted metabolism of DMT by ADMET Predictor®

The metabolic profile of 5-MeO-MiPT was also predicted (Table 7). 5-MeO-MiPT was predicted to be a substrate but not inhibitor for CYP1A2 and CYP2C9, a substrate for cytochromes P450 2A6 (CYP2A6) and 2B6 (CYP2B6). The compound is not expected to be a substrate for cytochrome P450 2E1 (CYP2E1). 5-MeO-DIPT is predicted to be a substrate but not inhibitor for CYP1A2 and CYP2C9, a substrate for CYP2B6 and CYP2E1, while it is not predicted to be a substrate for CYP2A6. DMT is expected to be a substrate but not inhibitor for CYP1A2 and CYP2C9 and a substrate for CYP2A6 and CYP2E1. The three compounds were predicted to be both substrates and inhibitors for cytochrome P450 2D6 (CYP2D6). None of the compounds was predicted to be inhibitor or substrate for cytochrome P450 3A4 (CYP3A4). ADMET predicted the metabolic profile of 5-MeO-MiPT in human liver microsomes (HLM). ADMET predictor showed that 5-MeO-MiPT could produce four main metabolites (Fig. 9): N-deisopropyl metabolite (M1); N-demethyl metabolite (M2); O-demethyl metabolite (M3); and 1-OH-isopropyl metabolite (M4). On the other hand, two metabolites from 5-MeO-DIPT are estimated to be produced (Fig. 10): N-demethyl metabolite (M1) and O-demethyl metabolite (M2). Lastly, DMT is expected to be metabolized to (Fig. 11): N-oxide metabolite (M1), 5-OH metabolite (M2), and N-demethyl metabolite (M3).

**Table 7:** Predicted cytochrome enzymes involved in 5-MeO-MiPT, 5-MeO-DIPT, and DMT metabolism and their percentage of accuracy (%) by ADMET Predictor®

DRUG	CYP 3A4		CYP 1A2		CYP 2C9		CYP 2A6		CYP 2B6		CYP 2D6		CYP2E1	
	Inh	Subs	Inh	Subs	Inh	Subs	Inh	Subs	Inh	Subs	Inh	Subs	Inh	Subs
5-MeO-MiPT	No (90%)	No (84%)	No (97%)	Yes (91%)	No (99%)	Yes (66%)	**–**	Yes (40%)	**–**	Yes (64%)	Yes (70%)	Yes (87%)	**–**	No (73%)
5-MeO-DIPT	No (90%)	No (84%)	No (97%)	Yes (91%)	No (99%)	Yes (50%)	**–**	No (70%)	**–**	Yes (77%)	Yes (70%)	Yes (87%)	**–**	Yes (66%)
DMT	No (90%)	No (84%)	No (97%)	Yes (91%)	No (99%)	Yes (39%)	**–**	Yes (66%)	**–**	No (56%)	Yes (55%)	Yes (87%)	**–**	Yes (59%)

## Discussion

This study shows that the 5-Meo-MIPT identified in the seizures of law enforcement and responsible for the intoxication of a human subject causes relevant pharmaco-toxicological effects in the preclinical model.

### Behavioural tests

#### Evaluation of visual response (object and placing response tests)

The manifestation of pharmacological effects after the administration of 5-MeO-MiPT was dose-dependent. Our results first showed that 5-MeO-MiPT induces a decrease of response to visual stimulation that has been seen through both object and placing response tests. In the visual object response test, 5-MeO-MiPT starts to have an effect at the 1 mg/kg dose, while it immediately reduces the visual placing starting from the 0.1 mg/kg. This trend of the visual response has also been described by our work group after the administration of LSD in mice (Tirri et al. 2022). The effect on visual functionality can be explained with the presence of 5HT_2A_ receptors in primary visual cortex (V1) of mammals. In this regard, a study conducted on macaque demonstrated a modulatory effect of 5HT_2A_ and serotonin receptor 1B (5HT_1B_) that depends on level of neuronal response. Activation of 5HT_1B_ caused a facilitation of visual response from V1 high-firing neurons and suppressed low-firing ones. Administration of DOI, a 5-HT_2A_ agonist, induced an opposite modulatory effect, suppressing the response from the high-firing neurons and facilitating responses from low-firing ones. Therefore, it is thought that 5HT_2A_ receptor has a gain control in response to visual stimulation (Watakabe et al. 2009). Moreover, 5HT_2A_ receptors’ role in reduction of visual response has been demonstrated in murine model. In fact, it has been showed that the administration of DOI induces a decrease in visual response and the surround suppression in primary visual cortex (Michaiel et al. 2019).

#### Evaluation of overall tactile and acoustic responses

The effect of hyperstimulation of the overall tactile response observed after the injection of the high doses (10–30 mg/kg) of 5-MeO-MiPT is in accordance with our previous preclinical studies conducted with LSD (Tirri et al. 2022), while the effect of 5-MeO-MiPT seems to be opposite to the one observed after the injection of DOB, that induces an inhibition of tactile response that may be caused by the stimulation of 5HT_2A_ receptors in the cortical areas of the brain (Tirri et al. 2020). Regarding acoustic response, our results suggest that 5-MeO-MiPT at the doses tested do not induce significant effects.

#### Evaluation of core temperature and body surface temperature

Concerning thermoregulation, 5-MeO-MiPT causes a significant hypothermic effect only at the highest dose (30 mg/kg) and in a transient manner. Its effect deviates from LSD, which induces a dose-dependent increase of core temperature (Holze et al. 2022a). A study conducted on thermoregulation induced by 36 different tryptamines in rat and rabbits showed that just few of them caused variation in core temperature. Among them, 5-MeO-DIPT induced hypothermia during the period of multiple administration in rats, but 24 h after the last injection hyperthermia occurred (Araújo et al. 2015). Thus, it cannot be excluded a similar profile of effect on body temperature for 5-MeO-MiPT and further studies are required to clarify this aspect. The fact that hallucinogens can alter thermoregulation is in accordance with the roles of serotonin in maintenance of body homeostasis: activation of _2_5HT_1_, 5HT_2_, 5HT_3_, and 5HT_7_ receptors participates to thermoregulation through different mechanisms, as O_2_ consumption, CO_2_ excretion, or heat loss (Ishiwata 2014; Barnes et al. 2021; Voronova 2021). Specifically, previous studies showed that central 5HT_1A_ receptor’s activation induces hypothermia in rats, affecting both heat production and heat loss processes. Contrary, central 5HT_2_ receptors have an opposite effect on rat body temperature, leading to hyperthermia. Also, it has been observed that peripheral 5HT_2_ receptors mediate the hypothermic effect described after the injection of 5HT in ICR mice (Voronova 2021). Moreover, it is been widely described the serotonin syndrome, a condition associated with drugs that alter the regulation of serotonin (such as monoamine oxidase inhibitors, MAOI, serotonin reuptake inhibitors, SSRI, or serotonin agonist), that, among all the symptoms, causes hyperthermia (Wang et al. 2016).

#### Stimulated motor activity assessment

Administration of 5-MeO-MiPT at the dose of 30 mg/kg significantly reduced the time spent on the rotarod and the number of steps performed by mice in a transient manner, suggesting that high doses of 5-MeO-MiPT produce an impairment of motor coordination. At date, no study on the effect on stimulated motor activity of tryptamines has been conducted but it is worth noting that designer tryptamines cause a decrease in spontaneous locomotor activity in rats (Krebs-Thomson et al. 2006; Williams et al. 2007; Pic-Taylor et al. 2015). Moreover, a similar effect has been observed in mice after the administration of psilocin and 5-MeO-DMT and it was demonstrated that the mechanism responsible for it is the activation of 5HT_1A_ receptors (Halberstadt et al. 2011). Also, it is important to highlight that one of the symptoms observed in the patient of the case we reported was catatonia, accordingly to previous investigations that have shown that hallucinogens, especially at high doses, can produce withdrawal and catatonia-like state (Halberstadt and Geyer 2013).

#### Startle/prepulse inhibition (PPI) analysis

Prepulse inhibition (PPI) describes a phenomenon where the response to a sensorial stimulus (startle) is attenuated when preceded by a weaker stimulus (prepulse; Halberstadt 2015). PPI is often used as a measure of sensorial gating and it is one of the most typical tests conducted to characterize hallucinogen compounds, which have been postulated to disrupt sensory filtering mechanism and to cause sensory overload and cognitive dysfunction (Halberstadt and Geyer 2018).

From our analysis, we found that 5-MeO-MIPT causes a dose-dependent impairment of sensorial gating, according to previous studies that demonstrated the inhibition of PPI in murine model as a result of administration of hallucinogens, such as LSD, DOB, 2C-B, and “-NBOMe” serie phenethylamines (Halberstadt 2015; Halberstadt and Geyer 2018; Tirri et al. 2022). To date, the mechanism by which 5-MeO-MiPT can alter PPI is still unknown and more studies are needed and different mechanisms might be involved. The impairment of sensorial gating induced in rats by LSD and DOI has been attributed to the activation of 5HT_2A_ receptors, because this effect is blocked by the 5HT_2_ antagonist M100907, but not by selective antagonists to subtypes 2B, 2C, and 1A (Halberstadt 2016). 5-MeO-DMT has also been found to disrupt PPI in rats, but the effect is primarily mediated by 5HT_1A_ and not by 5HT_2A_ receptors (Krebs-Thomson et al. 2006; Halberstadt 2016). Interestingly, it has been found that tryptamine hallucinogens have an opposite effect on PPI in mice. It has been shown that 5-MeO-DMT and psilocin dose-dependently increase PPI in mice and the effect of 5-MeO-DMT was partially attenuated by 5HT_1A_ selective antagonist WAY-100635 (Halberstadt and Geyer 2011).

The effect on sensory functionality we found in mice fits in well with the action of hallucinogen compounds on human; in fact, it is known that these substances alter the mechanisms implicated in the processing of sensorial and sensorimotor information of the cortex (Preller et al. 2019). Specifically, 5HT_2A_ receptors are expressed in the prefrontal cortex (PFC), which is involved in the sense of awareness of the external environment and in higher cognitive functions, and in the thalamus, where reticular nucleus regulates the flow of information between thalamus and cortex (Nichols 2016).

### Cardiorespiratory analysis

In addition to behavioural effects, we have demonstrated through our study that systemic administration of 30 mg/kg of 5-MeO-MiPT can alter cardiorespiratory parameters. In fact, it affects heart rate, inducing an initial tachycardia followed by a significant bradycardia and reappearance of tachycardia. It also causes a decrease in pulse distention and an alteration of the breath rate. Tachycardia was one of the symptoms of 5-MeO-MiPT of the intoxication case reported above and was also described by some users on online forum (Psychonaut 2011). BP-2000 analysis showed that the compound elicits an increase of both systolic and diastolic blood pressure after an initial fall. All these effects fall within the profile of tryptamine designer drugs which as previously mentioned, cause in human tachypnoea, hypertension, and tachycardia, and are in accordance with the ones evoked by severe classic psychedelics (Schlag et al. 2022), including psilocybin (Carbonaro et al. 2018; Holze et al. 2022b) and DMT (Strassman and Qualls 1994). Conversely, administration of 5-MeO-DMT in rats has been observed to induce bradycardia and a biphasic effect in blood pressure, with a hypotension preceded by a primary hypertension (Dabire et al. 1987). It is well known that serotonin has a variety of actions on cardiovascular (Kaumann and Levy 2006) and respiratory systems (Barnes et al. 2021) and mediates the neuronal regulation of blood pressure (Watts et al. 2012). Specifically, previous findings suggest that the stimulation of 5HT_1A_ induces central sympathoinhibition and decreases blood pressure, whereas 5HT_2A_ mediate sympathomimetic effects such as increased heart rate, vasomotor tone, and blood pressure (Villalón and Centurión 2007; Reckweg et al. 2022). As previous studies (Chaouche-Teyara et al. 1994; Buchborn et al. 2020) conducted on psychedelics suggest, the mechanism behind the effects of 5-MeO-MiPT on blood pressure may also involve the stimulation of peripheral serotonin receptors located on the vascular smooth muscle and the endothelium, by which 5HT acts as a direct vasopressor regulator (Frishman and Grewall 2000; Villalón and Centurión 2007).

### *In silico *ADMET prediction

Considering the lack of knowledge of the toxicokinetic effects of 5-MeO-MiPT, we decided to apply in silico ADMET prediction for 5-MeO-MiPT in comparison to 5-MeO-DIPT and DMT. The ADMET risk scores of the three compounds and their predicted pharmacokinetic properties were similar. They are shown to have a high permeability and to almost cross the BBB completely. In line with these findings, all the three compounds are very lipophilic; in fact, the drug permeability across membranes by passive diffusion is strongly related to its lipophilicity (Fan and de Lannoy 2014). Moreover, none of the drugs is predicted to be a substrate of P-gp, OAT1, and OAT3, which are efflux transporters expressed in the BBB and can limit the bioavailability of the drugs in the brain (Löscher and Potschka 2005; Nigam et al. 2015). In addition, they present high values of predicted Vd and their percentage unbound to blood plasma protein are over 30%. These data taken together reveal that the three compounds have a good ability to distribute and reach the target tissues (Zhang et al. 2012). Notably, only 5-MeO-DIPT and DMT are shown to have a potential risk code related to Vd, whereas 5-MeO-MiPT presents a lower predicted Vd and fits the threshold risk of ADMET (< 3.7 L/Kg).

The three compounds are not predicted to induce elevated levels of serum alanine transaminase (Ser_ALT), serum lactate dehydrogenase (Ser_LDH), serum alkaline phosphatase (Ser_AlkPhos), and serum gamma-glutamyl transferase (Ser_GGT) in human. An alteration of these hepatic enzymes can be associated to liver damages or alteration of the bile flow (Giannini et al. 2005). Moreover, 5-MeO-MiPT, 5-MeO-DIPT, and DMT are not predicted to inhibit the bile salt export pump, as the BSEP_IC50 predicted are beyond the threshold. Liver damage or alteration of the bile flow can be related to an alteration of hepatic enzyme levels (Giannini et al. 2005) and the inhibition of BSEP, which leads to an increase of intracellular bile salt concentration to toxic level, is one of the causes of drug induced liver injury (DILI; Kis et al. 2012). These evidences show that 5-MeO-MiPT, 5-MeO-DIPT, and DMT are not expected to induce hepatotoxicity. It is worth to note that Altuncı and colleagues previously conducted an in vivo experiment on female mice and found that high dosages of 5-MeO-MiPT may induce histopathological effects on liver, kidneys, and brain. Also, high doses of 5-MeO-MiPT caused an increase of levels of caspase-3 and caspase-8 in the tissues, suggesting that the compound could trigger apoptotic cell death (Altuncı et al. 2021). However, their findings are not in contradiction with our prediction; in fact, we estimated the hepatotoxic risk of the compound only in human and not in murine model. Finally, the primary clearance mechanism predicted for 5-MeO-MiPT, 5-MeO-DIPT, and DMT was metabolism and ADMET Predictor® highlights a cytochrome risk related to 5-MeO-MiPT and 5-MeO-DIPT. Specifically, 5-MeO-MiPT is exposed to high clearance by CYP1A2 and CYP2C9, while 5-MeO-DIPT only by CYP1A2. Moreover, all the three drugs are predicted to be both substrate and inhibitor to CYP2D6, an enzyme that takes part in metabolizing many commonly used drugs (Taylor et al. 2020) and psychoactive substances (Kirchheiner et al. 2011), and the inhibition of which could lead to drug-drug interaction.

The described metabolisms of 5-MeO-MiPT, 5-MeO-DIPT, and DMT involve reactions of N-dealkylation, O-demethylation, and hydroxylation of the parent drug. According to in silico ADMET prediction, 5-MeO-MiPT and DMT are both expected to be metabolized to 5-MeO-NIPT. These results are partially in line with a previous study conducted by Grafinger and colleagues, who analysed three samples from an intoxication case associated to 5-MeO-MiPT and proposed a possible metabolic pathway for the compound, using both in vivo and in vitro samples. Their study showed the production of 5-MeO-NIPT and 5-OH-MiPT in all the sample types (Grafinger et al. 2018). The proposed pathway is similar to the one described for 5-MeO-DIPT, and it involves CYP1A2, CYP2C19, CYP3A4, and CYP2D6 (Kamata et al. 2010).

The in silico ADMET prediction has already been used by our workgroup (Arfè et al. 2023) and it once again proved to be a useful tool in preliminary characterization of the pharmaco-toxicological effect on NPS.

## Conclusion

**Fig. 12:**
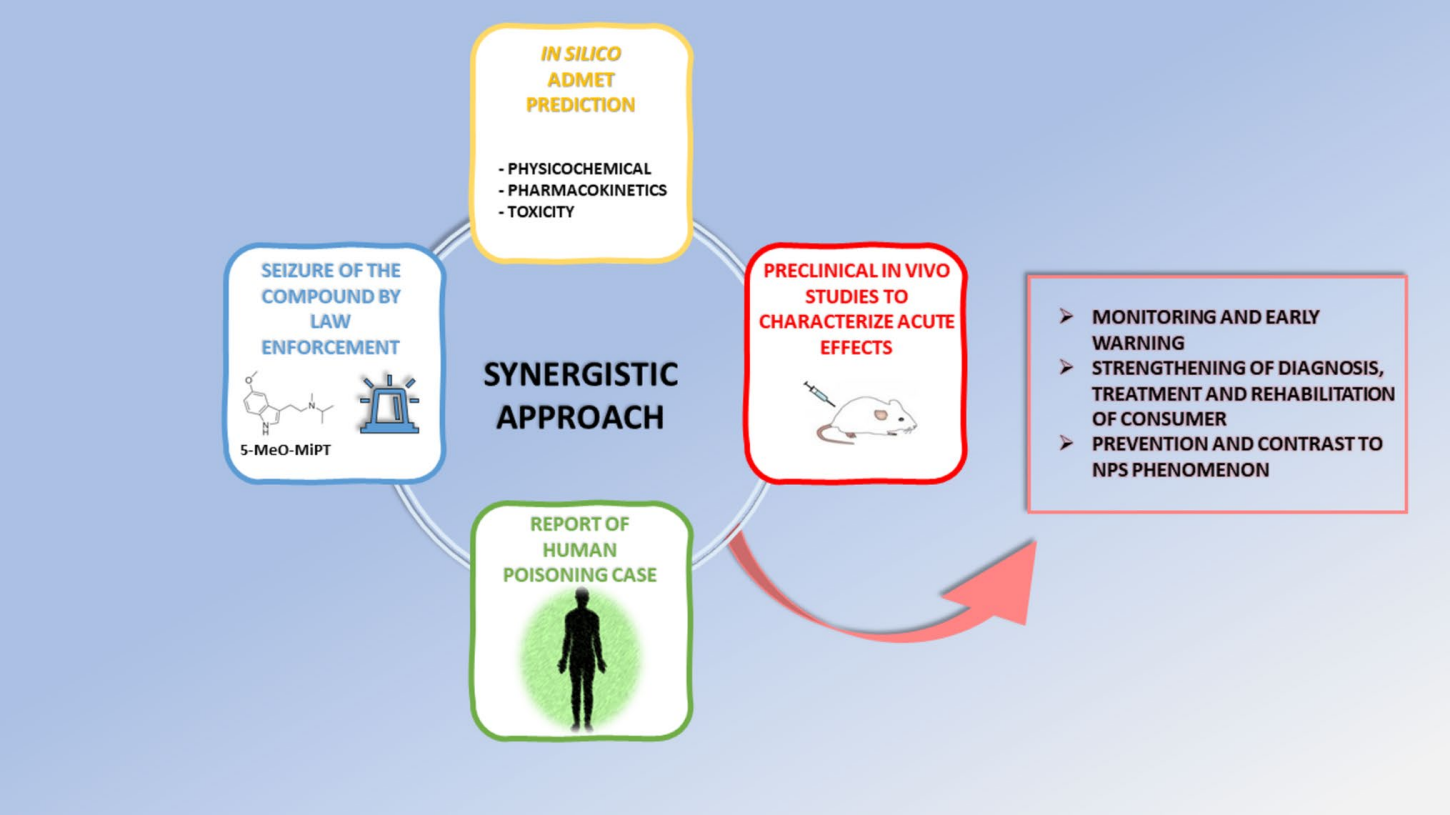
Schematic representation of the competency model for the pharmaco-toxicological evaluation (in vivo preclinical studies and in silico ADMET prediction) of the effects of NPS seized in the national territory and involved in human intoxications, as a result of the collaboration among the Police Forces (Arma of Carabinieri), the Pavia Poison Control Centre (PCC)—National Toxicology Information Centre, and the preclinical drug-toxicology laboratory (Collaborative Centre of DPA)

The present study is the result of a collaboration among the Police Forces (Arma of Carabinieri), the Pavia Poison Control Centre (PCC)—National Toxicology Information Centre, and the preclinical drug-toxicology laboratory (Collaborative Centre of DPA) and represents an integrated competence model (Fig. 12) for the pharmaco-toxicological evaluation (in vivo preclinical studies and in silico ADMET prediction) of the effects of NPS seized in the national territory, which can cause intoxications in humans.

The preclinical in vivo study demonstrates that 5-MeO-MiPT inhibits, in a dose-dependent manner, the visual response and the sensorial gating. Also, we demonstrated that high doses of the compound impair the motor coordination and induce cardiorespiratory alterations in mice. Moreover, we emphasize that the human intoxication case includes symptoms that match the effects evoked by 5-Meo-MiPT in mice, suggesting that 5-MeO-MiPT, among the other compounds found in blood and urine samples, played a role in the induction of the patient health status. In silico ADMET prediction revealed a cytochrome risk of 5-MeO-MiPT and the possible interaction with other substances by inhibiting CYP2D6. Worthily noting, the compound is not predicted to have a risk of hepatotoxicity. The exposure to high doses of this compound may lead to a risk due to its effects on cardiorespiratory system. Moreover, the psychomotor and sensory impairment together with the hallucinatory state that it induces can profoundly affect the daily activities of the consumer and, for example, have implications for driving safety. In this regard, driving under the influence of hallucinogens has been associated with risk-taking and impulsive behaviours such as speeding or disregarding traffic signals (Salas-Wright et al. 2021). On the other hand, we highlight that the heaviest effects we found in mice were induced by doses of 5-MeO-MiPT that strongly exceed the human dose commonly consumed in order to experience the hallucinogen effects and that so far, the compound has never been associated with fatal intoxication. In those circumstances, we tend to consider that the risk of 5-MeO-MiPT is limited to the assumption of very high dosages, the combination with other substances, or the intake of the substance under conditions requiring the full ability of the subject to perform his activities. Given that and the in silico ADMET profile obtained, we consider 5-MeO-MiPT a compound of which the therapeutic potential in the treatment of psychiatric disorders, such as depression, anxiety, post-traumatic stress disorder, addiction, and eating disorders, can be explored. In fact, in recent years there has been a revival of interest in psychedelics in this area and several studies and clinical trials have suggested their effectiveness in the treatment of alcohol dependence, anxiety, and major depression disorder (Bogenschutz et al. 2015; Dos Santos et al. 2016; Davis et al. 2021; De Gregorio et al. 2021).

## Abbreviations

4F-MPH: 4-Fluoromethylphenidate
5HT: Serotonin
5HT_1A_: Serotonin receptor 1A
5HT_1B_: Serotonin receptor 1B
5HT_2A_: Serotonin receptor 2A
5-MeO-DIPT: 5-Methoxy-N,N-diisopropyltryptamine
5-MeO-MiPT: 5-Methoxy-N-methyl-N-isopropyltryptamine
Absn_Risk: Absorption risk
ADMET: Absorption, distribution, metabolism, excretion, toxicity
ADMET_Risk: Absorption, distribution, metabolism, excretion, toxicity risk
BBB: Blood brain barrier
BSEP: Bile salt export pump
CYP_Risk: Cytochrome risk
CYP1A2: Cytochrome P450 1A2
CYP2A6: Cytochrome P450 2A6
CYP2B6: Cytochrome P450 2B6
CYP2C9: Cytochrome P450 2C9
CYP2E1: Cytochrome P450 2E1
CYP3A4: Cytochrome P450 3A4
CYPs: Cytochromes P450
DCK: Deschloroketamine
DET: N,N-diethyltryptamine
DMT: N,N-dimethyltryptamine
ECCS: Extended Clearance Classification System
Fup: Percent unbound to blood plasma proteins
hERG: Human voltage-dependent K + channel inhibition
HLM: Human liver microsomes
Hum_fup: Percent unbound to blood plasma proteins in human
Hum_RBP: Blood to plasma ratio in human
MDCK: Madin-Darby canine kidney
Mou_fup: Percent unbound to blood plasma proteins in mouse
Mou_RBP: Blood to plasma ratio in mouse
NET: Norepinephrine transporter
NPS: Novel psychoactive substances
OAT1: Organic anion transporter 1
OAT3: Organic anion transporter 3
Peff: Jejunal permeability
Pgp: P-glycoprotein
PPI: Prepulse inhibition
RBP: Blood to plasma ratio
S + CL_Mech: Clearance mechanism
Ser_AlkPhos: Serum alkaline phosphatase
Ser_ALT: Serum alanine transaminase
Ser_AST: Serum aspartic acid transaminase
Ser_GGT: Serum gamma-glutamyl transferase
Ser_LDH: Serum lactate dehydrogenase
SERT: Serotonin transporter
SNAP: National Early Warning System
SpO2: Oxygen blood saturation
TOX_Risk: Toxicity risk
Vd: Volume of distribution
